# Supplementary material for: Impact of maternal common mental disorders on child educational outcomes at 7 and 9 years: a population-based cohort study in Ethiopia
Source: BMJ Open. 2018 Jan 21;8(1):e018916. doi: 10.1136/bmjopen-2017-018916 (PMC5781096; doi:10.1136/bmjopen-2017-018916)
Supplement: Author's manuscript [file bmjopen-2017-018916.draft_revisions.pdf]

BMJ Open is committed to open peer review. As part of this commitment we make the peer review history of every article we publish publicly available.

When an article is published we post the peer reviewers' comments and the authors' responses online. We also post the versions of the paper that were used during peer review. These are the versions that the peer review comments apply to.

The versions of the paper that follow are the versions that were submitted during the peer review process. They are not the versions of record or the final published versions. They should not be cited or distributed as the published version of this manuscript.

BMJ Open is an open access journal and the full, final, typeset and author-corrected version of record of the manuscript is available on our site with no access controls, subscription charges or pay-per-view fees (http://bmjopen.bmj.com).

If you have any questions on BMJ Open's open peer review process please email editorial.bmjopen@bmj.com

Impact of maternal common mental disorders on child educational outcomes at 7 and 9 years: a population-based cohort study in Ethiopia

Journal:	*BMJ Open*
Manuscript ID	bmjopen-2017-018916
Article Type:	Research
Date Submitted by the Author:	01-Aug-2017
Complete List of Authors:	Mekonnen, Habtamu; Addis Ababa University, College of Health Sciences, School of Medicine, Department of Psychiatry<br>Medhin, Girmay; Addis Ababa University, Aklilu Lemma Institute of Pathobiology<br>Tomlinson, Mark; University of Stellenbosh,<br>Alem, Atalay; Addis Ababa University, College of Health Sciences, School of Medicine, Department of Psychiatry<br>Prince, Martin; King's College London, Institute of Psychiatry, Psychology and Neuroscience, King's Global Health Institute<br>Hanlon, Charlotte; Addis Ababa University, College of Health Sciences, School of Medicine, Department of Psychiatry; King's College London, Institute of Psychiatry, Psychology and Neuroscience, Centre for Global Mental Health
**Primary Subject Heading**:	Mental health
Secondary Subject Heading:	Epidemiology, Global health
Keywords:	MENTAL HEALTH, EPIDEMIOLOGY, Community child health < PAEDIATRICS

SCHOLARONE™ Manuscripts

Impact of maternal common mental disorders on child educational outcomes at 7 and 9 years: a population-based cohort study in Ethiopia

Habtamu Mekonnen[1], Girmay Medhin[3], Mark Tomlinson[4], Atalay Alem[1], Martin Prince[2] and Charlotte Hanlon[1, 2]

1. Department of Psychiatry, School of Medicine, College of Health Sciences, Addis Ababa University, Ethiopia;
2. Centre for Global Mental Health, Institute of Psychiatry, King's College London, UK;
3. Aklilu-Lemma Institute of Pathobiology, Addis Ababa University, Ethiopia;
4. Department of psychology, Stellenbosch University, Stellenbosch, South Africa.

Corresponding Author: Charlotte Hanlon, Department of Psychiatry, 6th Floor, College of Health Sciences Building, Tikur Anbessa Hospital, P. O. Box 9086, Addis Ababa, Ethiopia. Telephone: +251 912803374. E-mail: charlotte.hanlon@kcl.ac.uk

Word count: 3242

For peer review only

ABSTRACT

Objectives: To examine the association between maternal common mental disorders (CMD) and child educational outcomes. We hypothesised that children exposed to higher maternal CMD symptoms would have increased absenteeism and drop-out and poorer academic achievement over 24 months.

Design: A population-based cohort study (the CMaMiE study: child outcomes in relation to maternal mental health).

Setting: the study was undertaken in the Butajira Health and Demographic Surveillance Site, a predominantly rural area of Ethiopia, located 135 km south to the capital Addis Ababa.

Participants: Inclusion criteria were: aged between 15 and 49 years, able to speak Amharic, third trimester of pregnancy and resident of the Health and Demographic Surveillance site. Out of 1234 eligible women, 1065 women were recruited between July 2005 and February 2006 and followed up. When the child was 6.5 years old, the size of the cohort was expanded to include an additional 1345 mothers and children who had been born in the 12 months preceding and following the recruitment of the original cohort.

Primary and secondary outcome measures: Maternal CMD was measured using the Self-Reporting Questionnaire, validated for the setting. Educational outcomes (academic achievement, absenteeism, drop-out) of the child were obtained from school records when the children were aged 8 to 9 years and from the mother at the age of 7 to 8 years.

Result: After adjusting for potential confounders, maternal CMD at 7 to 8 years was associated significantly with school drop-out (Odds Ratio 1.07; 95% Confidence Interval (CI): 1.00, 1.13, P=

For peer review only - http://bmjopen.bmj.com/site/about/guidelines.xhtml

0.043) and absenteeism (Incidence Rate Ratio 1.01; 95% CI: 1.00, 1.02 P=0.026) at 8 to 9 years. There was no significant association between maternal CMD and child academic achievement.

Conclusion: Our findings are consistent with the expanding body of evidence indicating that interventions to improve maternal mental health may have benefits for child education.

Keywords: Child Education, Absenteeism, Maternal Mental Health, Depression, sub-Saharan Africa

Article summary: strengths and limitations of this study

• To the best of our knowledge this is the first study from a low-income country to investigate prospectively the association between maternal mental health and school absenteeism, drop-out and academic performance in the child.

• Methodological strengths include the representative, population-based sample and large sample size.

• We relied on non-standardised tests of child academic achievement and on routinely collected data to measure absenteeism, which may have led to non-differential misclassification and under-estimation of the strength of association.

What is already known on this subject?

Maternal common mental disorder (CMD), particularly depression, is associated with poorer child growth, health, development and survival during early childhood. The effect of exposure to maternal CMD during pre-and early school years upon the subsequent educational outcomes of the child has been little investigated in low-income country settings.

For peer review only - http://bmjopen.bmj.com/site/about/guidelines.xhtml

For peer review only

What this study adds?

This prospective study has shown that children who exposed to maternal CMD during pre- and early school years have higher school absenteeism and drop-out in the subsequent year. Current global efforts to scale-up mental health care provide an opportunity to integrate mental health interventions into community programmes to improve child retention in education.

For peer review only

For peer review only - http://bmjopen.bmj.com/site/about/guidelines.xhtml

INTRODUCTION

Child education is a crucial component of holistic child development;[1] however, even when children have access to schooling, they may not fully use their potential. An early nurturing environment is integral to establishing the solid foundations essential for child development, wellbeing and later academic success. In a systematic review of studies from low- and middle-income countries (LMICs), common mental disorders (CMD: clinically significant depressive and anxiety symptoms) affected 15.6% of women during pregnancy and 19.8% during the postnatal period.[2] Evidence is accumulating from middle-income[3-5] and low-income[6-8] countries that the children of mothers with CMD have less optimal growth, cognitive and language development, even when taking social adversity into account. This association may be mediated through disruption of mother-child interactions[7, 9] as has been seen in high-income country settings.[10] In LMICs, maternal CMD has also been associated with low birth weight,[11] prolonged labour,[12] child ill-health[13] and child under-nutrition,[14, 15] all of which are established risk factors for poor child development.[16, 17] Despite this emerging evidence base, to the best of our knowledge, there have been just two previous studies investigating the impact of maternal CMD on child educational outcomes, both of which were conducted in Barbados, at the time an upper middle income country.[18, 19] There have been no published studies from rural LMIC settings or low-income countries.

In the current study, we examined the association between preschool and early school age exposure to maternal CMD and child educational outcomes in an ongoing population-based

For peer review only - http://bmjopen.bmj.com/site/about/guidelines.xhtml

cohort study of mothers and children in rural Ethiopia. We hypothesised that children exposed to high symptoms of maternal CMD would have poorer school attendance, higher school drop-out and poor academic achievement over 24 months of follow-up.

METHODOLOGY

Study Design

The C-MaMiE study (child outcomes in relation to maternal mental health in Ethiopia) is a population-based cohort study.[12]

Study Setting

This study was undertaken in Butajira, Gurage Zone, Southern Nations Nationalities and Peoples' Region of Ethiopia. Butajira is a predominantly rural area, located 135 km south of the capital Addis Ababa, within a Health and Demographic Surveillance Site (HDSS). The HDSS was established in 1986 under the Butajira Rural Health Programme,[20] with one urban and nine rural administrative sub-districts from different ecological zones. In each sub-district, there is an easily accessible health post (frontline primary care) and an elementary school. Mixed farming of cash crops like Khat (*Catha edulis Forsk*) and chilli peppers, and production of staples, like maize, "false banana" or Ensete (*Ensete ventricosun*), form the basis of the economy.

Context for Education

In Ethiopia, primary education lasts for eight years (age group 7-14 years) with two cycles: basic (grades 1-4) and general education (grades 5-8). In the first cycle, children are taught and evaluated by a single teacher following the 'self-contained class' concept.[21] Ethiopia is working

For peer review only - http://bmjopen.bmj.com/site/about/guidelines.xhtml

towards complete coverage of primary education, yet only 85.5% has been achieved with 9.9% drop-out and 7.3 % grade repetition nationally.[22] The official age for school enrolment is seven years, [21]. All government schools are, in principle, free for all Ethiopians; however, families are expected to cover the costs of school uniforms, food and exercise books.[21] Except for two national examinations (at the completion of grades 10 and 12) and one regional examination (at the completion of grade 8), the academic performance of students is assessed by the class teacher using non-standardized tests.

Study Participants

The C-MaMiE birth cohort was established in the Butajira HDSS between July 2005 and February 2006 to investigate the public health impact of perinatal CMD.[12] A population-based sample of 1065 women was recruited out of 1234 eligible women (86.3%) with inclusion criteria of ages between 15 and 49 years, ability to speak Amharic, resident of the HDSS and in the third trimester of pregnancy. The cohort of women and the child born from the index pregnancy have been assessed repeatedly over time. When the C-MaMiE project children were six and a half years of age, the cohort size was augmented by 1345 children and their mothers, (the 'expanded C-MaMiE cohort') who were identified by records in the HDSS as having been born in the 12 months preceding (n=572) and following (n=773) the recruitment of the C-MaMiE cohort.

Assessment time-points

Two assessments were conducted 12 months apart within the expanded C-MaMiE cohort; exposures were assessed when the children were aged 6 to 7 (6/7) years and 7 to 8 (7/8) years and educational outcomes when children were 7/8 years and 8/9 years old.

For peer review only - http://bmjopen.bmj.com/site/about/guidelines.xhtml

Measures

Outcomes

School drop-out: this was operationalised as the proportion of students who had enrolled at the beginning of the academic year (September) but who had dropped out of school before the end of the academic year (June) and was obtained from school records and each mother. Children who drop out of school can be re-enrolled for the subsequent academic year.

Academic Achievement: was assessed using the child's grade point averaged over the two semesters of the Ethiopian school year. Grade repetition occurs when the averaged grade point is <50%.

Absenteeism: was defined as the student missing school for a minimum of one day, irrespective of the reason given for the absence. In Ethiopia, each school keeps a daily attendance record for each student. For this study, the total number of days of absence from school was obtained from the school attendance sheet for the 2014/2015 academic year.

Primary exposure

Maternal CMD: was measured when the child was aged 6/7 and 7/8 years, using the World Health Organization (WHO) 20-item version of the Self-Reporting Questionnaire (SRQ-20).[23] The SRQ-20 asks about the existence or absence of depressive, anxiety and somatic symptoms in the preceding one month (answered ‘yes' or ‘no'). The SRQ-20 has been validated for perinatal women in this rural Ethiopian population.[24]

For peer review only - http://bmjopen.bmj.com/site/about/guidelines.xhtml

Potential confounding factors

The following potential confounding factors were measured at both the 6/7 and 7/8 year exposure time points:

Stressful life events: List of Threatening Experiences (LTE) measures 12 stressful life events over the preceding six months.[25] The LTE was translated into Amharic, adapted to local conditions and has been used in the C-MaMiE cohort study at all points of assessment.

Socio-economic status: was measured using self-report of current roof material, the experience of hunger in the preceding month due to lack of food or money, and the existence of emergency resources for times of crisis.

Paternal substance use: maternal report of frequency of alcohol or khat use by the father.

Demographic characteristics: age of the mother, marital status, literacy level, birth order and sex of the child were obtained from self-report of the mother when the children were 6/7. The updated marital status of the woman was also recorded when the children were 7/8 years. The HDSS records were used to calculate the age of the child.

Potential mediators

Child nutritional status: anthropometric measures were carried out by trained project data collectors. Weight was measured with digital floor scales. A stadiometer with a movable head piece was used for height. Using the WHO reference population,[26] height-for-age z scores were calculated using WHO Anthro software.[27]

Data Management

Data Collection Procedure: Interviews with the women and anthropometric measures of the child were carried out in the woman's home, or surrounding area, according to the woman’s

For peer review only - http://bmjopen.bmj.com/site/about/guidelines.xhtml

preference, to ensure privacy and confidentiality. Anthropometric measures were also conducted in school, when convenient. The project data collectors have all completed dipiloma level education and have been employed by the C-MaMiE project for the last 11 years. They are experienced in conducting interviews and in the use of the study measures. All data collectors received an additional three days of intensive training on the use of the instruments. The questionnaires were piloted before commencing data collection and discrepancies in ratings were discussed to ensure that the data collectors had a common understanding.

Maintaining data quality: Supervisors and a field coordinator monitored the data collection process and performed quality checks on a random sample of evaluations. Data were double entered with EpiData[28] by experienced data entry clerks on the day of data collection, where possible. Any identifiable information about the respondent was kept securely and separately detached from the assessment data and a code number was used to ensure confidentiality.

Statistical Analyses

The analysis was conducted using Stata software version 12.[29] A hypothesis-driven analysis was conducted to examine the association between maternal CMD (total score on the SRQ-20) and educational outcomes, guided by the conceptual model shown in Figure 1. Initially, univariate analyses were conducted: logistic regression for school drop-out and repetition of the year (binary), poisson regression for absenteeism (continuous, count data) and linear regression for academic achievement (continuous, normally distributed). Multivariable analyses were then conducted, adjusted for all potential confounders identified *a priori*. Child nutritional status was modelled separately to explore mediation of the association between maternal CMD and child educational outcomes. Estimates of association were presented with their corresponding 95%

For peer review only - http://bmjopen.bmj.com/site/about/guidelines.xhtml

confidence intervals. Complete case analysis was used. The study has been reported according to the STROBE reporting checklist.

ETHICAL CONSIDERATIONS

Ethical approval was obtained from the Institutional Review Board of the College of Health Sciences, Addis Ababa University, Ethiopia (reference number 082/13/psy) and the Research Ethics Committee of King's College London, UK (reference number PNM/13/14-92). Consent from each woman and assent from the child were obtained. Any woman who presented with high symptoms of mental health problems and suicidal ideation was advised to seek care at the psychiatric unit at Butajira Hospital, with the project covering treatment and transportation costs.

RESULTS

A total of 2090 mother-child dyads were assessed at 6/7 years, and 1957 were assessed at 7/8 years. Those where were lost to follow-up did not differ in terms of demographic, socioeconomic and other background characteristics. There was no significant difference between those children who did and who did not have educational information available regarding maternal CMD, socioeconomic status, nutritional status or other background characteristics. See Figure 2.

See Tables 1 and 2 for the distribution of maternal and child characteristics across educational outcomes.

For peer review only - http://bmjopen.bmj.com/site/about/guidelines.xhtml

Table 1: Characteristics of participants in relation to drop-out from school

Characteristics		Child school drop-out by 7/8 years		Child school drop-out by 8/9 years	
		Dropped out	Attending School	Dropped out	Attending school
		N (%)	N (%)	N (%)	N (%)
		336 (20.2)	1326 (79.8)	153 (11.8)	1140 (88.2)
**Primary exposure**					
Maternal CMD	High SRQ-20 (6 or more)	19 (5.7)	54 (4.1)	20 (13.7)	117 (10.3)
**Parental characteristics**					
Maternal Age in years (Mean, Standard Deviation)		35 (6.00)	34 (6.00)	35 (6.00)	34 (6.00)
Literacy (mother)	Non-literate	307 (91.6)	1100 (83.3)	142 (93.4)	997 (88.0)
Literacy (father)	Non-literate	148 (46.7)	423 (33.3)	62 (42.5)	400 (36.8)
Maternal marital status	Monogamous	263 (78.5)	1089 (82.4)	116 (76.8)	908 (81.0)
	Polygamous	54 (16.1)	182 (13.8)	28 (18.5)	160 (14.3)
	Divorced, widowed, separated	18 (5.4)	50 (3.8)	7 (4.6)	53 (4.7)
**Socioeconomic status**					
Current hunger	Had hunger	42 (12.5)	80 (6.1)	12 (7.8)	50 (4.4)
Emergency resources	No emergency resources	164 (48.9)	485 (36.7)	49 (32.2)	322 (28.4)
Roof material	Thatched roof	242 (72.2)	810 (61.3)	94 (61.8)	601 (53.0)
**Psychosocial characteristics**					
Paternal substance use	Weekly khat use	264 (83.5)	976 (76.9)	104 (72.2)	880 (82.7)
	Weekly alcohol use	55 (17.4)	185 (14.6)	30 (20.8)	173 (16.2)
Negative life event in the last 6 months	No life event	272 (81.9)	1065 (81.2)	108 (71.1)	860 (75.9)
	1 life event	48 (14.5)	184 (14.0)	31(20.4)	196 (17.3)
	2 or more	12 (3.6)	63 (4.8)	13 (8.6)	77 (6.8)
**Child characteristics**					
Childbirth order	First	26 (7.7)	212 (16.0)	16 (10.5)	156 (13.7)
	Middle or last	310 (92.3)	1114 (84.0)	136 (89.5)	977 (86.2)
Sex of the child	Boy	201 (59.8)	670 (50.6)	85 (55.4)	590 (52.0)
Child nutritional status	Stunted (Height-for-age z-score < -2)	111 (33.4)	297 (22.6)	46 (30.3)	261 (23.2)

CMD = Common mental disorders; SRQ = Self-Reporting Questionnaire;

For peer review only - http://bmjopen.bmj.com/site/about/guidelines.xhtml

Table 2: Characteristics of participants and child absenteeism, academic achievement and repetition of the school year

Characteristics		Child School absenteeism by the age of 8/9 years	Child Academic achievement by the age of 8/9 years	Child grade repetition status by the age of 8/9 years	
		Median (25th, 75th percentiles)	Mean (SD)	Class Repeated	Promoted to next grade
		5 (2, 11)	62.5 (9.21)	N (%)	N (%)
				25 (2.4)	1029 (97.6)
**Primary exposure**					
Maternal CMD	High SRQ-20 (≥6)	2 (5, 11)	62.0 (9.54)	3 (12.0)	107 (10.5)
**Parental characteristics**					
Maternal age in years (mean (SD))			-	34 (6.00)	34 (6.00)
Literacy (mother)	Non-literate	2 (5, 12)	62.5 (9.29)	22 (88.0)	900 (87.9)
Literacy (father)	Non-literate	3 (6, 14)	61.3 (9.23)	13 (54.2)	351 (35.8)
Maternal marital status	Monogamous	2 (5, 12)	62.8 (9.34)	19 (76.0)	824 (81.4)
	Polygamous	2 (4, 9)	61.1 (8.91)	5 (20.0)	140 (13.8)
	Divorced, widowed, separated	1 (4, 6)	62.1 (8.13)	1 (4.0)	48 (4.7)
**Socioeconomic status**					
Current hunger	Had hunger	2 (5, 11)	62.1 (7.75)	1 (4.0)	44 (4.3)
Emergency resource	Had no emergency resource	2 (5, 11)	62.1 (9.07)	9 (36.0)	288 (28.1)
Roof materials	Thatched	3 (6, 14)	62.8 (9.65)	17 (68.0)	524 (51.2)
**Psychosocial characteristics**					
Paternal substance use	Weekly khat use	2 (5, 12)	62.3 (9.24)	16 (66.7)	791 (82.4)
	Weekly alcohol use	1 (3.5, 7)	64.6 (9.42)	6 (25.0)	150 (15.6)
Negative life event in the last six months	No life event	2 (5, 12)	62.5 (9.14)	20 (80.0)	775 (75.6)
	1 life event	2 (4, 9)	62.8 (9.84)	3 (12.5)	180 (17.6)
	2 or more	2 (3, 8)	61.2 (8.21)	2 (8.0)	68 (6.6)
**Child characteristics**					
Childbirth order	First	2 (4.5, 14)	62.8 (8.87)	5 (20.0)	136 (13.3)
	Middle or last	2 (5, 11)	62.5 (9.26)	20 (80.0)	887 (86.7)
Sex of the child	Boy	2 (5, 12)	62.6 (9.23)	15 (60.0)	528 (51.6)
Child nutritional status	Stunted (Height-for-age z-score < -2)	2 (5, 12)	62.5 (9.20)	6 (24.0)	233 (23.0)

CMD = Common mental disorders; SRQ = Self-Reporting Questionnaire; SD= standard deviation

For peer review only

For peer review only - http://bmjopen.bmj.com/site/about/guidelines.xhtml

The prevalence of maternal CMD was 5.2% when the children were 6/7 years and 10.9% when 7/8 years of age. The difference was statistically significant. School drop-out was 20.2% (n=336) at the 7/8 years and 11.8% (n=153) at the 8/9 years. At the 8/9 years, the median number of days of absence was 5 (interquartile range 2 - 11), the mean average grade point was 62.5 out of 100 (Standard Deviation 9.21) and 2.4 % of the children had repeated the year.

Controlling for potential confounders, maternal CMD at 7/8 years was associated significantly with child school drop-out (OR 1.07, 95% CI: 1.00, 1.13) at 8/9 years, but the significant univariate association between maternal CMD at 6/7 years and school drop-out by 7/8 years became non-significant (adjusted OR 1.05, 95% CI: 0.99, 1.12). In the fully adjusted multivariable model, parent non-literacy, low socioeconomic status, male gender, not being first in the birth order and lower height-for-age were associated significantly with school drop-out by the age of 7/8 years. At 8/9 years follow-up, paternal khat use and lower child height-for-age were associated significantly with school drop-out. Maternal CMD at 7/8 years was associated with absenteeism by 8/9 years (adjusted Incidence Rate Ratio 1.01, 95% CI: 1.00, 1.02). Absenteeism was also associated significantly with maternal age, parent non-literacy, widowed or separated marital status, low socioeconomic status, mother experiencing negative life event, child male gender, and being first in the birth order. See Tables 3 and 4.

For peer review only - http://bmjopen.bmj.com/site/about/guidelines.xhtml

Table 3: Factors associated with child school dropout by 7/8 and 8/9 years

Explanatory variables	Explanatory variables at 6/7 years and school drop-out as an outcome by age 7/8 years Odds Ratio (95% Confidence Interval)		Explanatory variables at 7/8 years and school drop-out as an outcome by age 8/9 years Odds Ratio (95% Confidence Interval)	
	Crude	Adjusted §	Crude	Adjusted §
**Primary exposure**				
Maternal CMD (SRQ-20 total score)	**1.07 (1.02 ,1.13)**	1.05 (0.99 , 1.12)	**1.07 (1.01,1.13)**	**1.07 (1.00, 1.13)**
**Other explanatory variables**				
Maternal age (years)	**1.03 (1.01, 1.05)**	1.02 (0.99, 1.03)	1.01 (0.98, 1.04)	1.01 (0.97, 1.05)
Non-literate mother	**2.20 (1.46, 3.33)**	**1.77 (1.13, 2.77)**	1.94 (0.99, 3.76)	1.64 (0.82, 3.27)
Non-literate father	**1.75 (1.37, 2.25)**	**1.53 (1.17, 2.01)**	1.26 (0.89, 1.78)	1.19 (0.82, 1.74)
Maternal marital status (polygamous, divorced and widowed)	1.08 (0.99, 1.18)	1.07 (0.82, 1.43)	1.03 (0.91, 1.18)	1.18 (0.78, 1.78)
Experienced hunger due to lack of resources	**2.22 (1.50, 3.29)**	**1.74 (1.10, 2.77)**	1.85 (0.96, 3.56)	1.78 (0.82, 3.84)
No emergency resources	**1.65 (1.30, 2.11)**	**1.43 (1.10, 1.89)**	1.20 (0.83, 1.72)	1.05 (0.69, 1.60)
Thatched roof	**1.65 (1.27, 2.14)**	1.30 (0.96, 1.74)	**1.43 (1.01, 2.03)**	1.33 (0.91, 1.96)
Paternal khat use at least weekly	**1.52 (1.10, 2.11)**	1.38 (0.98, 1.95)	**0.54 (0.37, 0.81)**	**0.52 (0.34, 0.79)**
Paternal alcohol use at least weekly	1.23 (0.89, 1.71)	1.35 (0.95, 1.93)	1.35 (0.88, 2.09)	1.18 (0.73, 1.89)
Negative life event (≥1 in the last 6 months)	0.99 (0.84, 1.16)	0.85 (0.69, 1.03)	1.04 (0.98, 1.89)	1.00 (0.77, 1.29)
Male child	**1.45 (1.14, 1.85)**	**1.50 (1.15, 1.96)**	1.15 (0.82, 1.62)	1.13 (0.78, 1.63)
Child first in birth order	**0.44 (0.29, 0.67)**	**0.59 (0.36, 0.95)**	0.73 (0.43, 1.27)	0.82 (0.45, 1.51)
Height for age z score	**0.73 (0.65, 0. 83)**	**0.80 (0.70, 0.91)**	**0.75 (0.62, 0.89)**	**0.73 (0.60, 0.89)**

CMD = common mental disorder; SRQ = Self-Reporting Questionnaire;

§ Parental characteristics including (maternal age, marital status, maternal and paternal level of literacy), Socio-economic status (hunger due to lack of resources, emergency resources, roof material), paternal substance use, negative life event, child sex, birth order and child nutritional status

For peer review only - http://bmjopen.bmj.com/site/about/guidelines.xhtml

Table 4: Association of factors by 7/8 years with child school absenteeism at 8/9 years

Explanatory variables	School absenteeism by 8/9years Incidence Rate Ratio (95% Confidence Interval)	
	Crude	Adjusted §
**Primary exposure**		
Maternal CMD (SRQ-20 total score)	1.00 (0.99, 1.01)	**1.01 (1.00, 1.02)**
**Other explanatory variables**		
Increasing maternal age (years)	1.00 (0.99, 1.01)	**1.01 (1.01, 1.02)**
Mother non literate	**1.60 (1.48, 1.74)**	**1.57 (1.44, 1.72)**
Father non literate	**1.18 (1.13, 1.23)**	**1.07 (1.02, 1.12)**
Marital status (polygamous, divorced, widowed)	**0.92 (0.89, 0.94)**	**0.88 (0.83, 0.93)**
Experienced hunger due to lack of resources	1.06 (0.96, 1.17)	1.06 (0.94, 1.19)
No emergency resources	1.03 (0.99, 1.08)	0.98 (0.94, 1.04)
Thatched roof (vs. corrugated iron)	**1.46 (1.40, 1.53)**	**1.36 (1.30, 1.42)**
Father uses khat at least weekly	**1.10 (1.03, 1.17)**	1.04 (0.97, 1.11)
Father uses alcohol at least weekly	**0.90 (0.85, 0.96)**	0.92 (0.86, 0.98)
Negative life event (≥1 in the last 6 months)	**0.98 (0.97, 0.99)**	**0.90 (0.87, 0.94)**
Male child	1.04 (1.00, 1.09)	**1.05 (1.00, 1.09)**
Child first in birth order	**1.14 (1.08, 1.21)**	**1.17 (1.10, 1.25)**
Height for age z score	1.00 (0.97, 1.01)	1.00 (0.97, 1.02)

CMD = common mental disorder; SRQ = Self-Reporting Questionnaire;

§ Parental characteristics including (maternal age, marital status, maternal and paternal level of literacy), Socio-economic status (hunger due to lack of resources, emergency resources, roof material), paternal substance use, negative life event, child sex, birth order and child nutritional status

For peer review only - http://bmjopen.bmj.com/site/about/guidelines.xhtml

There was no significant association between maternal CMD and academic achievement or grade repetition. Paternal non-literacy, alcohol use and widowed or separated maternal marital status were significantly associated independently with these outcomes. See Table 5.

For peer review only

For peer review only - http://bmjopen.bmj.com/site/about/guidelines.xhtml

Table 5: Association of other factors at 7/8 years with child educational outcomes by 8/9 years

Explanatory variables	Academic achievement by 8/9 years β coefficient (95% Confidence Interval)		Grade repetition by 8/9 years Odds Ratio (95% Confidence Interval)	
	Crude	Adjusted §	Crude	Adjusted §
Primary exposure				
Maternal CMD (SRQ-20 total score)	-0.07 (-0.27, 0.13)	0.01 (-0.22, 0.23)	1.01 (0.87, 1.16)	0.99 (0.85, 1.16)
Other explanatory variables				
Maternal age	-0.03 (-0.13, 0.08)	0.01 (-0.11, 0.13)	1.01 (0.94, 1.09)	1.05 (0.96, 1.14)
Mother non-literate	0.21 (-1.51, 1.92)	0.46 (-1.42, 2.34)	1.00 (0.29, 0.97)	0.87 (0.24, 3.17)
Father non- literate	**-1.88 (-3.06, -0.69)**	**-1.90 (-3.16, -0.64)**	2.12 (0.93, 4.78)	1.89 (0.80, 4.44)
Marital status (polygamous, divorced and widowed)	-0.21 (-0.67, 0.26)	**-2.02 (-3.48, -0.55)**	0.96 (0.67, 1.38)	1.31 (0.54, 3.16)
Experienced hunger due to lack of resources	-0.42 (-3.18, 2.33)	-0.79 (-4.14, 2.56)	0.93 (0.12, 7.00)	0.97 (0.11, 8.74)
No emergency resources	-0.59 (-1.83, 0.64)	-0.59 (-2.00, 0.80)	1.43 (0.63, 3.28)	1. 50 (0.60, 3.74)
Thatched roof	0.54 (-0.57, 1.66)	0.78 (-0.46, 2.00)	2.02 (0.86, 4.73)	1.55 (0.62, 3.89)
Father uses khat at least weekly	-1.31 (-2.82, 0.21)	-0.57 (-2.15, 1.00)	0.43 (0.18, 1.01)	0.44 (0.18, 1.12)
Father uses alcohol at least weekly	**2.47 (0.88, 4.05)**	**2.35 (0.65, 4.04)**	1.80 (0.70, 4.62)	1. 26 (0.45, 3.51)
Negative life event in the last six months	-0.07 (-0.33, 0.18)	-0.24 (-1.09, 0.61)	1.04 (0.90, 1.18)	0.76 (0.34, 1.72)
Male child	0.16 (-0.95, 1.28)	-0.09 (-1.10, 1.27)	1.41 (0.63, 3.16)	1.45 (0.61, 3.42)
Child first in birth order	0.34 (-1.30, 1.97)	0.33 (-1.50, 2.17)	1.63 (0.60, 4.41)	2.09 (0.66, 6.60)
Height for age z score	**0.62 (0.00, 1.23)**	0.59 (-0.07, 1.25)	1.08 (0.69, 1.67)	1.12 (0.69, 1.81)

CMD = common mental disorder; SRQ = Self-Reporting Questionnaire;

§ Parental characteristics including (maternal age, marital status, maternal and paternal level of literacy), Socio-economic status (hunger due to lack of resources, emergency resources, roof material), paternal substance use, negative life event, child sex, birth order and child nutritional status

For peer review only - http://bmjopen.bmj.com/site/about/guidelines.xhtml

When child nutritional status was removed from the multivariable model, the association between maternal CMD and child educational outcomes was not strengthened at either time point, indicating the absence of mediation.

DISCUSSION

We found a significant prospective association between maternal CMD and school drop-out and absenteeism. The association did not appear to be mediated by nutritional status of the child. Parental low socioeconomic status, non-literacy and substance use were associated significantly with adverse educational outcomes of children. There was no association between maternal CMD and child academic achievement or grade repetition.

As far as we are aware, our study is the first of its kind from a low-income country to report the association between exposure to preschool maternal CMD and subsequent adverse educational outcomes. Strengths of the study included the population-based design, large sample size, high follow-up rates and use of culturally validated measures. Nonetheless, there were limitations of our study. We assessed academic achievement using a composite and non-standardised measure (individual teacher assessment). Although this approach increased measurement error, composite measures which include participation, homework, attendance and tests given by teachers may be more ecologically valid and tied to day-to-day routine of teaching and learning than narrowly focused assessments of content mastery. Absenteeism was extracted from data collected routinely by schools, which is likely to have led to underestimation of absences, but this would have led to a non-differential error which would be expected to

For peer review only - http://bmjopen.bmj.com/site/about/guidelines.xhtml

reduce the chance of finding a significant association and would not be expected to have led to bias. We relied on proxy indicators of socioeconomic status. Although these indicators have been developed for the population under study, they relied on self-report from the women and may not have been sufficiently comprehensive, thus raising the possibility of residual confounding.

Maternal CMD was more prevalent at the 7/8 year time point compared to 6/7 years. This difference might have arisen because the 7/8 years time point was measured during the rainy season, a time of hardship and food shortage, while the 6/7 time point was distributed over the year according to the birth date of the child. The non significant association between maternal CMD and academic achievement and grade repetition in our study is in contrast to the study from Barbados, where postpartum depression was associated with poorer academic achievement at 11 years of age.[19] This difference may have arisen due to the measures of academic achievement used (standardized in Barbados vs. subjective/composite measure in Ethiopia) and the timing of the CMD exposure (postpartum vs. pre- and early school years). Furthermore, the broader context for learning also differs substantially between Barbados and Ethiopia. In Ethiopia, the majority of women are non-literate and there is limited availability of learning resources (e.g. books, games) within the home. Given the critical importance of these factors for child learning, any additional effect of maternal CMD may have been too small to detect, unlike in Barbados.

For peer review only

On the other hand, the deficits in the home learning environment mean that regular attendance at school becomes the most important way for a child to learn and is likely to have greater influence on child educational performance. Our findings were in keeping with the Barbados study, where depressive symptoms in mothers of a child with an early history of malnutrition were associated with school absences between 5 and 11 years of age. Irregular school attendance is likely to be on the pathway to poorer educational attainment and to identify a group of children who are at risk of fully dropping out from school.

In studies from HICs, erratic school attendance and drop-out from school have been associated with an adverse early home environment, living with smokers, the quality of early care-giving, socioeconomic status, cognitive development, behavior problems, academic achievement, peer relations and parent involvement.[30, 31] In LMICs, food insecurity and death of parents[32, 33] are associated with irregular school attendance and dropping out entirely. In both HIC and LMIC studies, maternal CMD could be an important unmeasured mediator of the effects of adverse home environment on school attendance. For children under the age of ten years, absenteeism and drop-out are unlikely to happen without the knowledge of the family, and in particular the mother.

School absenteeism and drop-out have important economic implications, particularly in a low-income country like Ethiopia. Repeating years of education or students taking longer to complete their education bring inefficiency into the school system, financial burden on the household and may adversely affect the motivation of the child to pursue their education. For

For peer review only - http://bmjopen.bmj.com/site/about/guidelines.xhtml

those students who drop out of school altogether, there are economic losses from negative impacts on future productivity. Every additional year of schooling has a substantial positive effect on adult wages.[34] Achieving high coverage of primary school enrolment is undermined by subsequent early drop-out from schooling, which will limit Ethiopia's ability to deliver on the Sustainable Development Goal (SDG) target to ensure that all girls and boys complete free, equitable and quality primary and secondary education by 2030.

School-based interventions targeting specific predictors of absenteeism and drop-out in LMICs, e.g. deworming,[35] hand wash campaigns, safe water and hygiene,[36] and school feeding programmes,[37] have been found to be effective in reducing absenteeism and drop-out. Our study indicates that interventions (at family, school or community level) focusing on the reduction of absenteeism and drop-out need also to incorporate maternal CMD. There is a global impetus to improve access to primary care-based mental health care in LMICs through the World Health Organisation's mental health Gap Action Programme.[38] Evidence-based treatment packages for maternal depression that can be delivered by general health workers (assessment and prescription of medication) and lay workers (psychosocial approaches) have been shown to be effective, feasible and acceptable to communities across LMIC settings.[39] Effective care for maternal depression has been shown to have beneficial effects on child health in Pakistan[40] but further studies are needed to evaluate the impact on child educational outcomes.

For peer review only - http://bmjopen.bmj.com/site/about/guidelines.xhtml

CONCLUSION

In this population-based cohort study in rural Ethiopia, children of mothers with symptoms of common mental disorders were at greater risk of school drop-out and absenteeism. Future studies are needed to understand the mechanisms underlying this association. Current global efforts to expand access to mental health care offer an opportunity to address maternal CMD as a key component of programmes to increase school attendance.

Acknowledgements

We are grateful for the women who participated in the study for giving their time and energy to complete interviews and to the staff in the schools who facilitated data collection process. Our thanks also go to the project data collectors and staff of the C-MaMiE project in Butajira.

Contributors

HM, CH, AA and MP conceptualised the study. HM led data collection with oversight from CH, AA and GM. HM led data analysis, with input from CH, AA, MP, MT and GM. HM drafted the manuscript and all other co-authors revised it critically. All authors approved the final version of the paper and agree to be accountable for all aspects of the work.

Funding

The funding for the study came from the Wellcome Trust (project grant: 093559).

For peer review only - http://bmjopen.bmj.com/site/about/guidelines.xhtml

Competing interests

The funding for the study came from the Wellcome Trust.

Ethical approval

Ethical approval was obtained from the Institutional Review Board of the College of Health Sciences, Addis Ababa University, Ethiopia (reference number 082/13/psy) and the Research Ethics Committee of King's College London, UK (reference number PNM/13/14-92).

Data sharing statement

The data are being used for a PhD student (HM) for their thesis and are not, therefore, available at the present time to the general public. The data may be requested from the corresponding author for verification of the analyses in this paper.

Figure 1: Conceptual framework

Figure 2: Follow-up chart for child educational outcomes

For peer review only - http://bmjopen.bmj.com/site/about/guidelines.xhtml

REFERENCES

1. United Nations. Convention on the Rights of the Child. Geneva: UN1989.
2. Fisher J, Cabral de Mello M, Patel V, Rahman A, Tran T, Holton S et al. Prevalence and determinants of common perinatal mental disorders in women in low- and lower-middle-income countries: a systematic review:. Bull World Health Organ ;90:139–149G | doi:102471/BLT11091850:. 2012.
3. Galler JR, Harrison RH, Ramsey F, Forde V, Butler SC. Maternal depressive symptoms affect infant cognitive development in Barbados. Journal of Child Psychology & Psychiatry & Allied Disciplines. 2000;41(6):747-57.
4. Patel V, DeSouza N, Rodrigues M. Postnatal depression and infant growth and development in low income countries: a cohort study from Goa, India. Arch Dis Child. 2003;88(1):34-7.
5. Black MM, Baqui AH, Zaman K, Arifeen SE, Black RE. Maternal depressive symptoms and infant growth in rural Bangladesh. The American Journal of Clinical Nutrition. 2009;89(3):951S-7S. doi:10.3945/ajcn.2008.26692E.
6. Harpham T, Huttly S, De Silva MJ, Abramsky T. Maternal mental health and child nutritional status in four developing countries. Journal of Epidemiology and Community Health. 2005;59:1060-4.
7. Cooper PJ, Tomlinson M, Swartz L, Landman M, Molteno C, Stein A et al. Improving quality of mother-infant relationship and infant attachment in socioeconomically deprived community in South Africa: randomised controlled trial. Bmj. 2009;338:b974. doi:http://dx.doi.org/10.1136/bmj.b974.
8. Servili C, Medhin G, Hanlon C, Tomlinson M, Worku B, Baheretebeb Y et al. Maternal common mental disorders and infant development in Ethiopia: the P-MaMiE Birth Cohort. BMC Public Health. 2010;10:693.
9. Cooper PJ, Tomlinson M, Swartz L, Woolgar M, Murray L, Molteno C. Post-partum depression and the mother-infant relationship in a South African peri-urban settlement. Br J Psychiatry. 1999;175:554-8.
10. Murray L, Fiori-Cowley A, Hooper R, Cooper P. The impact of postnatal depression and associated adversity on early mother-infant interactions and later infant outcome. Child Dev. 1996;67(5):2512-26.
11. Ferri CP, Mitsuhiro SS, Barros MC, Chalem E, Guinsburg R, Patel V et al. The impact of maternal experience of violence and common mental disorders on neonatal outcomes: a survey of adolescent mothers in Sao Paulo, Brazil. BMC Public Health. 2007;7(1):209. doi:10.1186/1471-2458-7-209.
12. Hanlon C, Medhin G, Alem A, Tesfaye F, Lakew Z, Worku B et al. Impact of antenatal common mental disorders upon perinatal outcomes in Ethiopia: the P-MaMiE population-based cohort study. Tropical medicine & international health : TM & IH. 2009;14(2):156-66. doi:10.1111/j.1365-3156.2008.02198.x.
13. Rahman A, Iqbal Z, Bunn J, Lovel H, Harrington R. Impact of maternal depression on infant nutritional status and illness: a cohort study. Arch Gen Psychiatry. 2004;61(9):946-52.
14. Adewuya AO, Ola BO, Aloba OO, Mapayi BM, Okeniyi JAO. Impact of postnatal depression on infants' growth in Nigeria. Journal of Affective Disorders. 2008;108(1-2):191-3.
15. Surkan P, Kennedy. C, Hurley. K, Black. M. Maternal depression and early childhood growth in developing countries: systematic review and meta-analysis. Bull World Health Organ 2011;287:607-15.

For peer review only - http://bmjopen.bmj.com/site/about/guidelines.xhtml

16. Ramchandani P, Stein A, Evans J, O'Connor GT. Paternal depression in the postnatal period and child development: a prospective population study. Lancet. 2005;365:2201-5.
17. Parsons CE, Young SK, Rochat JT, Kringelbach LM, Stein A. Postnatal depression and its effects on child development: a review of evidence from low- and middle-income countries. British Medical Bulletin. 2012;101:57-79.
18. Salt P, Galler JR, Ramsey CF. The influence of early malnutrition on subsequent behavioural development. VII. The effect of maternal depressive symptoms. Developmental and Behavioural Pedatrics. 1988;9(1):1-5.
19. Galler JR, Ramsey FC, Harrison RH, Taylor J, Cumberbatch G, Forde V. Postpartum maternal moods and infant size predict performance on a national high school entrance examination. Journal of Child Psychology & Psychiatry. 2004;45(6):1064-75.
20. Berhane Y, Wall S, Kebede D, Emmelin A, Enquoselassie F, Byass P et al. Establishing an epidemiological field laboratory in rural areas - potentials for public health research and interventions (Special Issue). Ethiopian Journal of Health Development. 1999;13.
21. Federal Democratic Government of Ethiopia Ministry of Education. The education and training policy and its implementation, Ethiopia. Addis Ababa, Ethiopia,2002.
22. Federal Democratic Government of Ethiopia Ministry of Education. Education Statistics 2007 E.C. (2014/2015). Addis Ababa, Ethiopia: MoE, EMIS and ICT Directorate,2016.
23. Beusenberg M, Orley J. A user's guide to the Self Reporting Questionnaire (SRQ). Geneva: World Health Organization1994.
24. Hanlon C, Medhin G, Alem A, Araya M, Abdulahi A, Hughes M et al. Detecting perinatal common mental disorders in Ethiopia: validation of the self-reporting questionnaire and Edinburgh Postnatal Depression Scale. J Affect Disord. 2008;108(3):251-62. doi:10.1016/j.jad.2007.10.023.
25. Brugha TS, Bebbington PE, Tennant C, Hurry J. The list of threatening experiences: a subset of 12 life event categories with considerable long-term contextual threat. Psychological Medicine. 1985;15:189-94.
26. WHO Expert Committee. Physical status: the use and interpretation of anthropometry. Geneva: WHO1995.
27. World Health Organization. Anthro software version 3.2.2. http://www.who.int/childgrowth/software/en/. Geneva: WHO; 2011.
28. Lauritsen JM, Bruus M. Epidata (Version 3). A comprehensive tool for validated entry and documentation of data. . Odense, Denmark,: The Epidata Association,; 2003.
29. Stata Corporation. Stata Statistical Software Release 12. College Station, TX,: Stata Corporation; 2007.
30. Jimerson S, Egeland B, Sroufe LA, Carlson B. A prospective longitudinal study of high school dropouts: examining multiple predictors across development. Journal of School Psychology. 2000;38(6):525-49.
31. Levy DE, Winickoff JP, Rigotti NA. School absenteeism among children living with smokers. Pediatrics. 2011;128(4):650-6.
32. Case A, Ardington C. The impact of parental death on school outcomes: longitudinal evidence from South Africa. Demography. 2006;43(3):401-20.
33. Belachew T, Hadley C, Lindstrom D, Gebremariam A, Lachat C, Kolsteren P. Food insecurity, school absenteeism and educational attainment of adolescents in Jimma Zone Southwest Ethiopia: a longitudinal study. Nutrition Journal. 2011;10:29.

For peer review only - http://bmjopen.bmj.com/site/about/guidelines.xhtml

34. Day JC, Newburger EC. The big payoff: educational attainment and synthetic estimates of work-life earnings. Special studies. . Washington, DC,: Bureau of the Census (DOC), Economics and Statistics Administration2002.
35. Aiken MA, Davey C, Hargreaves RJ, Hayes JR. Re-analysis of health and educational impacts of a school-based deworming programme in western Kenya: a pure replication. International Journal of Epidemiology. 2015:1572-80 doi: 10.093/ije/dyv127.
36. O'Reilly CE, Freeman MC, Ravani M, Migele J, Mwaki A, Ayalo M et al. The impact of a school-based safe water and hygiene programme on knowledge and practices of students and their parents: Nyanza Province, western Kenya, 2006. Epidemiology and infection. 2008;136(1):80-91. doi:10.1017/s0950268807008060.
37. King N, Dewey C, Borish D. Determinants of primary school non-enrollment and absenteeism: results from a retrospective, convergent mixed methods, cohort study in rural western Kenya. PloS one. 2015;10(9):e0138362. doi:10.1371/journal.pone.0138362.
38. World Health Organization. mhGAP: Mental Health Gap Action Programme: scaling up care for mental, neurological, and substance use disorders. Geneva: WHO2008.
39. World Health Organization. mhGAP Intervention Guide version 2.0 for mental, neurological and substance use disorders in non-specialized health settings. Geneva: WHO; 2016.
40. Maselko J, Sikander S, Bhalotra S, Bangash O, Ganga N, Mukherjee S et al. Effect of an early perinatal depression intervention on long-term child development outcomes: follow-up of the Thinking Healthy Programme randomised controlled trial. The Lancet Psychiatry. 2015;2(7):609-17. doi:10.1016/S2215-0366(15)00109-1.

For peer review only

For peer review only - http://bmjopen.bmj.com/site/about/guidelines.xhtml

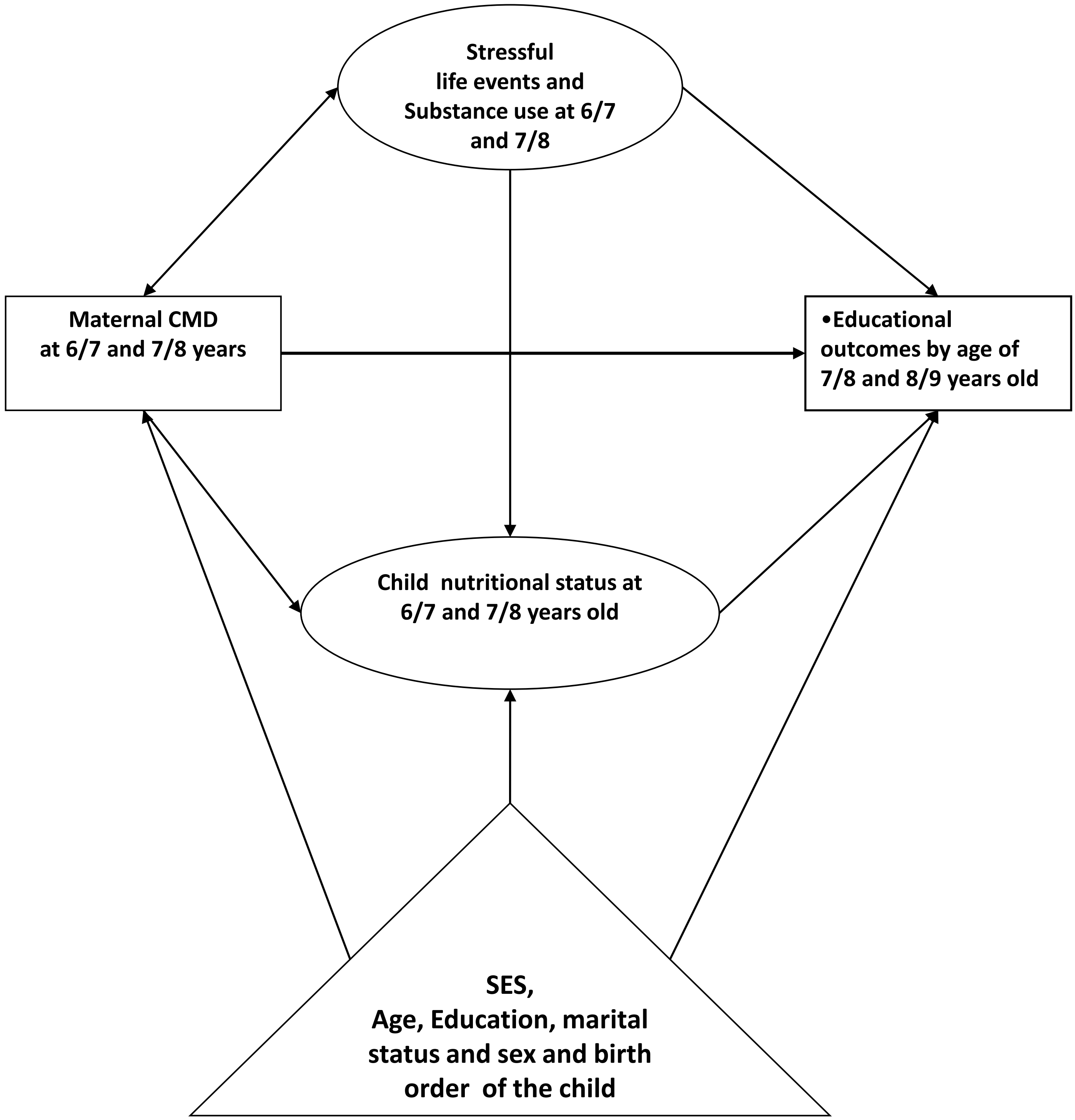
Stressful
life events and
Substance use at 6/7
and 7/8
Maternal CMD
at 6/7 and 7/8 years
•Educational
outcomes by age of
7/8 and 8/9 years old
Child nutritional status at
6/7 and 7/8 years old
SES,
Age, Education, marital
status and sex and birth
order of the child

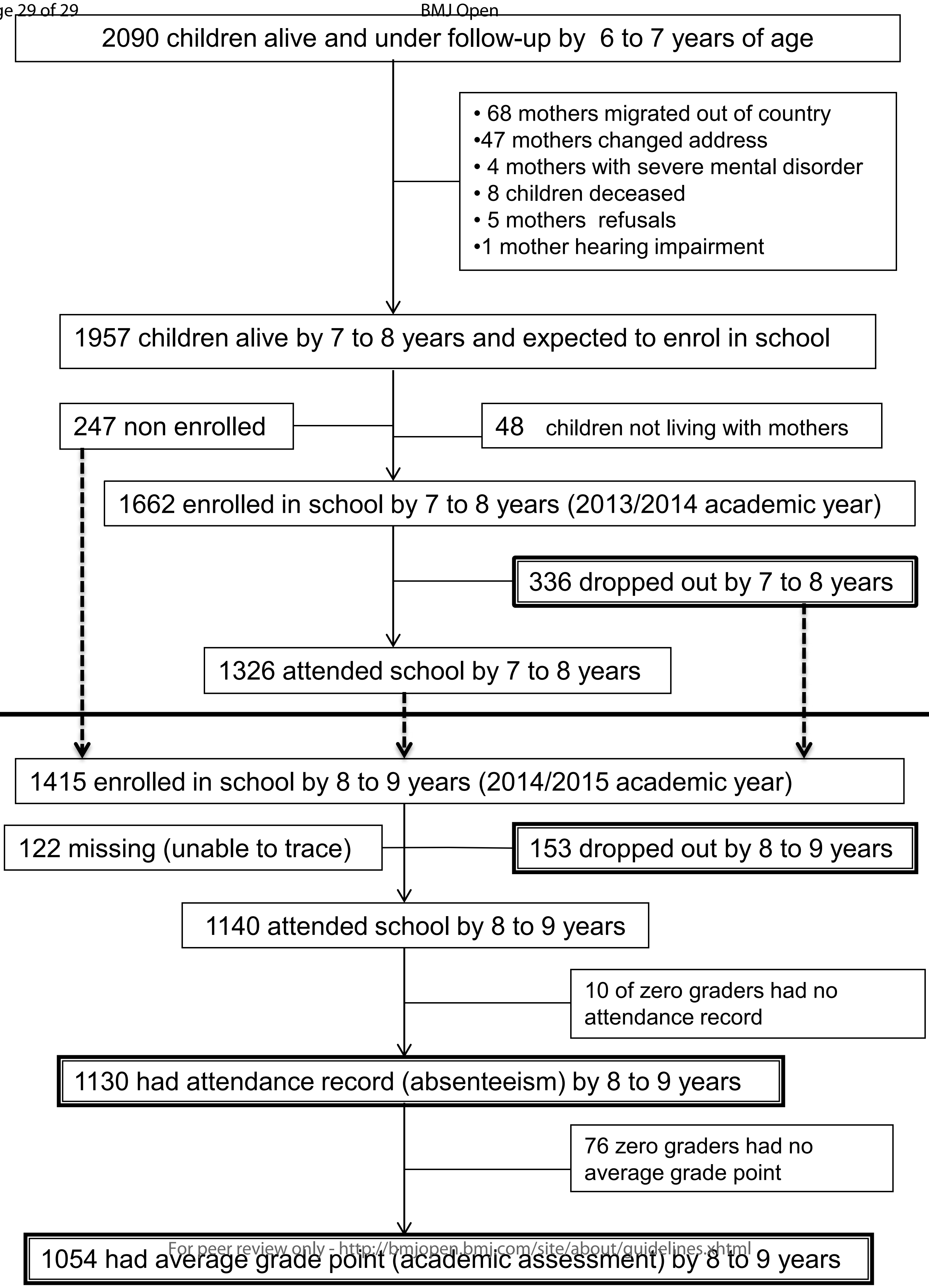
2090 children alive and under follow-up by 6 to 7 years of age
• 68 mothers migrated out of country
•47 mothers changed address
• 4 mothers with severe mental disorder
• 8 children deceased
• 5 mothers refusals
•1 mother hearing impairment
1957 children alive by 7 to 8 years and expected to enrol in school
247 non enrolled
48 children not living with mothers
1662 enrolled in school by 7 to 8 years (2013/2014 academic year)
336 dropped out by 7 to 8 years
1326 attended school by 7 to 8 years
1415 enrolled in school by 8 to 9 years (2014/2015 academic year)
122 missing (unable to trace)
153 dropped out by 8 to 9 years
1140 attended school by 8 to 9 years
10 of zero graders had no attendance record
1130 had attendance record (absenteeism) by 8 to 9 years
76 zero graders had no average grade point
1054 had average grade point (academic assessment) by 8 to 9 years

BMJ Open

Impact of maternal common mental disorders on child educational outcomes at 7 and 9 years: a population-based cohort study in Ethiopia

Journal:	*BMJ Open*
Manuscript ID	bmjopen-2017-018916.R1
Article Type:	Research
Date Submitted by the Author:	20-Oct-2017
Complete List of Authors:	Mekonnen, Habtamu; Addis Ababa University, College of Health Sciences, School of Medicine, Department of Psychiatry<br>Medhin, Girmay; Addis Ababa University, Aklilu Lemma Institute of Pathobiology<br>Tomlinson, Mark; University of Stellenbosh,<br>Alem, Atalay; Addis Ababa University, College of Health Sciences, School of Medicine, Department of Psychiatry<br>Prince, Martin; King's College London, Institute of Psychiatry, Psychology and Neuroscience, King's Global Health Institute<br>Hanlon, Charlotte; Addis Ababa University, College of Health Sciences, School of Medicine, Department of Psychiatry; King's College London, Institute of Psychiatry, Psychology and Neuroscience, Centre for Global Mental Health
**Primary Subject Heading**:	Mental health
Secondary Subject Heading:	Epidemiology, Global health
Keywords:	MENTAL HEALTH, EPIDEMIOLOGY, Community child health < PAEDIATRICS

SCHOLARONE™
Manuscripts

Impact of maternal common mental disorders on child educational outcomes at 7 and 9 years: a population-based cohort study in Ethiopia

Habtamu Mekonnen[1], Girmay Medhin[3], Mark Tomlinson[4], Atalay Alem[1], Martin Prince[2] and Charlotte Hanlon[1, 2]

1. Department of Psychiatry, School of Medicine, College of Health Sciences, Addis Ababa University, Ethiopia;
2. Centre for Global Mental Health, Institute of Psychiatry, King's College London, UK;
3. Aklilu-Lemma Institute of Pathobiology, Addis Ababa University, Ethiopia;
4. Department of psychology, Stellenbosch University, Stellenbosch, South Africa.

Corresponding Author: Charlotte Hanlon, Department of Psychiatry, 6th Floor, College of Health Sciences Building, Tikur Anbessa Hospital, P. O. Box 9086, Addis Ababa, Ethiopia. Telephone: +251 912803374. E-mail: charlotte.hanlon@kcl.ac.uk

Word count: 3610

For peer review only

ABSTRACT

Objectives: To examine the association between exposure to maternal common mental disorders (CMD) in pre- and early school age children and subsequent child educational outcomes.

Design: A population-based cohort study.

Setting: the study was undertaken in the Butajira Health and Demographic Surveillance Site (HDSS), a predominantly rural area of south central Ethiopia.

Participants: Inclusion criteria: women aged between 15 and 49 years, able to speak Amharic, in the third trimester of pregnancy and resident of the HDSS. 1065 women were recruited between July 2005 and February 2006 and followed up. When the average age of children was 6.5 years old, the cohort was expanded to include an additional 1345 mothers and children who had been born in the 12 months preceding and following the recruitment of the original cohort, identified from the HDSS records. Data from a total of 2090 mother-child dyads was included in the current analysis.

Measures: Maternal CMD was measured when the children were 6 to 7 ("6/7") and 7 to 8 ("7/8") years old using the Self-Reporting Questionnaire, validated for the setting. Educational outcome (drop-out) of the children at age 7/8 years (end of 2013/14 academic year) were obtained from maternal report. At age 8/9 years (end of 2014/15 academic year) educational outcomes (academic achievement, absenteeism, and drop-out) of the children were obtained from school records.

Result: After adjusting for potential confounders, exposure to maternal CMD at 7/8 years was associated significantly with school drop-out (Odds Ratio 1.07; 95% Confidence Interval (CI):

For peer review only - http://bmjopen.bmj.com/site/about/guidelines.xhtml

1.00, 1.13, P= 0.043) and absenteeism (Incidence Rate Ratio 1.01; 95% CI: 1.00, 1.02 P=0.026) at the end of 2014/15 academic year. There was no association between maternal CMD and child academic achievement.

Conclusion: Future studies are needed to evaluate whether interventions to improve maternal mental health can reduce child school absenteeism and drop-out.

For peer review only

Keywords: Child Education, Absenteeism, Maternal Mental Health, Depression, sub-Saharan Africa

For peer review only - http://bmjopen.bmj.com/site/about/guidelines.xhtml

Article summary: strengths and limitations of this study

• To the best of our knowledge this is the first study from a low-income country to investigate prospectively the association between maternal mental health and school absenteeism, drop-out and academic performance in the child.

• Methodological strengths include the representative, population-based sample and large sample size.

• We relied on non-standardised tests of child academic achievement and on routinely collected data to measure absenteeism, which may have led to non-differential misclassification and under-estimation of the strength of association.

- Diagnostic measures of common mental disorders would have been preferable to use of a screening scale.
- Chronic physical health problems in the child were not measured.

For peer review only

For peer review only - http://bmjopen.bmj.com/site/about/guidelines.xhtml

INTRODUCTION

Child education is a crucial component of holistic child development;[1] however, even when children have access to schooling, they may not fully use their potential. An early nurturing environment is integral to establishing the solid foundations essential for child development, wellbeing and later academic success. Maternal common mental disorders (CMD), characterized by depressive, anxiety and somatic symptoms, may compromise early child development.[2] In a systematic review of studies from low- and middle-income countries (LMICs), maternal CMD affected 15.6% of women during pregnancy and 19.8% during the postnatal period.[3] Evidence is accumulating from middle-income[4-6] and low-income[7-9] countries that the children of mothers with CMD have less optimal growth, cognitive and language development, even when taking social adversity into account. This association may be mediated through disruption of mother-child interactions[8, 10] as has been seen in high-income country settings.[11] In LMICs, maternal CMD has also been associated with low birth weight,[12] prolonged labour,[13] child ill-health[14] and child under-nutrition,[15, 16] all of which are established risk factors for poor child development.[17, 18] Despite this emerging evidence base, to the best of our knowledge, there have been just two previous studies investigating the impact of maternal CMD on child educational outcomes, both of which were conducted in Barbados, at the time an upper middle income country.[19, 20] There have been no published studies from rural LMIC settings or low-income countries.

In the current study, we examined the association between preschool and early school age exposure to maternal CMD and child educational outcomes in an ongoing population-based

For peer review only

cohort study of mothers and children in rural Ethiopia. We hypothesised that children exposed to high symptoms of maternal CMD would have poorer school attendance, higher school drop-out and poor academic achievement over 24 months of follow-up.

METHODOLOGY

Study Design

The C-MaMiE study (child outcomes in relation to maternal mental health in Ethiopia) is a population-based cohort study.[13]

Study Setting

This study was undertaken in Butajira, Gurage Zone, Southern Nations Nationalities and Peoples' Region of Ethiopia. Butajira is a predominantly rural area, located 135 km south of the capital Addis Ababa, within an HDSS. The HDSS was established in 1986 under the Butajira Rural Health Programme,[21] with one urban and nine rural administrative sub-districts from different ecological zones. In each sub-district, there is an easily accessible health post (frontline primary care) and an elementary school. Mixed farming of cash crops like Khat (*Catha edulis Forsk*) and chilli peppers, and production of staples, like maize, "false banana" or Ensete (*Ensete ventricosun*), form the basis of the economy.

Context for Education

In Ethiopia, primary education lasts for eight years (age group 7-14 years) with two cycles: basic (grades 1-4) and general education (grades 5-8). In the first cycle, children are taught and evaluated by a single teacher following the 'self-contained class' concept.[22] Ethiopia is working

For peer review only - http://bmjopen.bmj.com/site/about/guidelines.xhtml

towards complete coverage of primary education, yet only 85.5% has been achieved with 9.9% drop-out and 7.3 % grade repetition nationally.[23] The official age for school enrolment is seven years, [22]. All government schools are, in principle, free for all Ethiopians; however, families are expected to cover the costs of school uniforms, food and exercise books.[22] In the study area private schools are only found in the urban (Butajira town) district, with fewer than 0.3% of study children enrolled in these schools. Except for two national examinations (at the completion of grades 10 and 12) and one regional examination (at the completion of grade 8), the academic performance of students is assessed by the class teacher using non-standardized tests.

Study Participants

The C-MaMiE birth cohort was established in the Butajira HDSS between July 2005 and February 2006 to investigate the public health impact of perinatal CMD.[13] A population-based sample of 1065 women was recruited out of 1234 eligible women (86.3%) with inclusion criteria of ages between 15 and 49 years, ability to speak Amharic, resident of the HDSS and in the third trimester of pregnancy. Fewer than 3% of women were excluded on the basis of language at baseline. The cohort of women and the child born from the index pregnancy have been assessed repeatedly over time. When the average age of the C-MaMiE project children was six and a half years, the cohort size was augmented by 1345 children and their mothers, (the ‘expanded C-MaMiE cohort') who were identified by records in the HDSS as having been born in the 12 months preceding (n=572) and following (n=773) the recruitment of the C-MaMiE cohort. The same eligibility criteria were applied to the expanded cohort participants. In the current study, 2090 mother–child dyads remain under follow-up and participated in the study.

For peer review only - http://bmjopen.bmj.com/site/about/guidelines.xhtml

Assessment time-points

Two assessments were conducted 12 months apart within the expanded C-MaMiE cohort; exposures were assessed first when the children were aged 6/7 years and repeated at age 7/8 years. Drop-out of the children at age 7/8 years (end of 2013/14 academic year) was obtained from maternal report, while at age 8/9 years (end of 2014/15 academic year) the educational outcomes of absenteeism, drop-out and academic achievement were obtained from school records.

Measures

Outcomes

School drop-out: this was operationalised as the proportion of students who had enrolled at the beginning of the academic year (September) but who had dropped out of school before the end of the academic year (June) and was obtained from school records and each mother. Children who drop out of school can be re-enrolled for the subsequent academic year.

Academic Achievement: was assessed using the child's grade point averaged over the two semesters of the Ethiopian school year. Grade repetition occurs when the averaged grade point is <50%.

Absenteeism: was defined as the student missing school for a minimum of one day, irrespective of the reason given for the absence. In Ethiopia, each school keeps a daily attendance record for each student. For this study, the total number of days of absence from school was obtained from the school attendance sheet for the 2014/2015 academic year.

For peer review only - http://bmjopen.bmj.com/site/about/guidelines.xhtml

Primary exposure

Maternal CMD: was measured when the child was aged 6/7 and 7/8 years, using the World Health Organization (WHO) 20-item version of the Self-Reporting Questionnaire (SRQ-20).[24] The SRQ-20 is a screening tool which asks about the existence or absence of depressive, anxiety and somatic symptoms in the preceding one month (answered 'yes' or 'no'). The SRQ-20 has been validated for perinatal women in this rural Ethiopian population.[25]

Potential confounding factors

The following potential confounding factors were measured at both the 6/7 and 7/8 year exposure time points:

Stressful life events: List of Threatening Experiences (LTE) measures 12 stressful life events over the preceding six months.[26] The LTE was translated into Amharic, adapted to local conditions and has been used in the C-MaMiE cohort study at all points of assessment.

Socio-economic status: was measured using self-report of current roof material (corrugated iron vs. thatch), the experience of hunger in the preceding month due to lack of food or money, and the existence of emergency resources for times of crisis.

Paternal substance use: maternal report of frequency of alcohol or khat use by the father.

Demographic characteristics: age of the mother, marital status, literacy level, birth order and sex of the child were obtained from self-report of the mother when the children were 6/7. The updated marital status of the woman was also recorded when the children were 7/8 years. The HDSS records were used to calculate the age of the child.

Child nutritional status: anthropometric measures were carried out by trained project data collectors. Weight was measured with digital floor scales. A stadiometer with a movable head piece was used for height. Using the WHO reference population,[27] weight-for-age z scores

For peer review only - http://bmjopen.bmj.com/site/about/guidelines.xhtml

(underweight) and height-for-age z scores (stunting) were calculated using WHO Anthro software.[28] However, there was collinearity between height and weight and, therefore, height-for-age was included in the final model as it has been argued that it is a better summary measure of cumulative undernutrition [29].

Data Management

Data Collection Procedure: Interviews with the women and anthropometric measures of the child were carried out in the woman's home, or surrounding area, according to the woman's preference, to ensure privacy and confidentiality. Anthropometric measures were also conducted in school, when convenient. The project data collectors have all completed dipiloma level education and have been employed by the C-MaMiE project for the last 11 years. They are experienced in conducting interviews and in the use of the study measures. All data collectors received an additional three days of intensive training on the use of the instruments. Data collectors were not aware of the objectives of the study and interpretation of the score on the measures. Furthermore, they were randomly selected to carry out follow-up visits to specific households and time-points were separated by at least six months, so the chance of bias due to recall of maternal CMD status was very low. The questionnaires were piloted before commencing data collection and discrepancies in ratings were discussed to ensure that the data collectors had a common understanding.

Maintaining data quality: Supervisors and a field coordinator monitored the data collection process and performed quality checks on a random sample of evaluations. Data were double entered with EpiData[30] by experienced data entry clerks on the day of data collection, where

For peer review only - http://bmjopen.bmj.com/site/about/guidelines.xhtml

possible. Any identifiable information about the respondent was kept securely and separately detached from the assessment data and a code number was used to ensure confidentiality.

Statistical Analyses

The analysis was conducted using Stata software version 12.[31] The data were summarized using mean, median and percentage. A hypothesis-driven analysis was conducted to examine the association between maternal CMD (total score on the SRQ-20) and educational outcomes, guided by the conceptual model shown in Figure 1. Initially, unadjusted analyses were conducted: logistic regression for school drop-out and repetition of the year (binary), Poisson regression for absenteeism (continuous, count data) and linear regression for academic achievement (continuous, normally distributed). Multivariable analyses were then conducted, adjusted for all potential confounders identified *a priori*. Estimates of association were presented with their corresponding 95% confidence intervals. Complete case analysis was used. The study has been reported according to the STROBE reporting checklist.

ETHICAL CONSIDERATIONS

Ethical approval was obtained from the Institutional Review Board of the College of Health Sciences, Addis Ababa University, Ethiopia (reference number 082/13/psy) and the Research Ethics Committee of King's College London, UK (reference number PNM/13/14-92). Consent from each woman and assent from the child were obtained. Any woman who presented with high symptoms of mental health problems and suicidal ideation was advised to seek care at the psychiatric unit at Butajira Hospital, with the project covering treatment and transportation costs.

For peer review only - http://bmjopen.bmj.com/site/about/guidelines.xhtml

RESULTS

A total of 2090 mother-child dyads were assessed at 6/7 years, and 1957 were assessed at 7/8 years. See Figure 2. Those who were lost to follow-up did not differ in terms of age, marital status, level of literacy, negative life event, socioeconomic, substance use or on their mental health status. There was no significant difference between those children who did and who did not have educational information available regarding maternal CMD, socioeconomic status, parental literacy, substance use, negative life event, nutritional status, gender and their birth order. .

See Tables 1 and 2 for the distribution of maternal and child characteristics across educational outcomes.

For peer review only

Table 1: Characteristics of participants in relation to child school drop-out

Exposure variables		Exposure variables at age of 6/7 years Child school drop-out at end of 2013/14 academic year		Exposure variables at age of 7/8 years and Child school drop-out at end of 2014/15 academic year	
		Dropped out	Attending School	Dropped out	Attending school
		N (%)	N (%)	N (%)	N (%)
		336 (20.2)	1326 (79.8)	153 (11.8)	1140 (88.2)
**Parental characteristics**					
Maternal Age in years (Mean, Standard Deviation)		35 (6.00)	34 (6.00)	35 (6.00)	34 (6.00)
Literacy (mother)	Non-literate	307 (91.6)	1100 (83.3)	142 (93.4)	997 (88.0)
Literacy (father)	Non-literate	148 (46.7)	423 (33.3)	62 (42.5)	400 (36.8)
Maternal marital status	Monogamous	263 (78.5)	1089 (82.4)	116 (76.8)	908 (81.0)
	Polygamous	54 (16.1)	182 (13.8)	28 (18.5)	160 (14.3)
	Divorced, widowed, separated	18 (5.4)	50 (3.8)	7 (4.6)	53 (4.7)
**Socioeconomic status**					
Current hunger	Had hunger	42 (12.5)	80 (6.1)	12 (7.8)	50 (4.4)
Emergency resources	No emergency resources	164 (48.9)	485 (36.7)	49 (32.2)	322 (28.4)
Roof material	Thatched roof	242 (72.2)	810 (61.3)	94 (61.8)	601 (53.0)
**Psychosocial characteristics**					
Paternal substance use	Weekly khat use	264 (83.5)	976 (76.9)	104 (72.2)	880 (82.7)
	Weekly alcohol use	55 (17.4)	185 (14.6)	30 (20.8)	173 (16.2)
Negative life event in the last 6 months	No life event	272 (81.9)	1065 (81.2)	108 (71.1)	860 (75.9)
	1 life event	48 (14.5)	184 (14.0)	31(20.4)	196 (17.3)
	2 or more	12 (3.6)	63 (4.8)	13 (8.6)	77 (6.8)
**Child characteristics**					
Childbirth order	First	26 (7.7)	212 (16.0)	16 (10.5)	156 (13.7)
	Middle or last	310 (92.3)	1114 (84.0)	136 (89.5)	977 (86.2)
Sex of the child	Boy	201 (59.8)	670 (50.6)	85 (55.4)	590 (52.0)
Child nutritional status	Stunted (Height-for-age z-score < -2)	111 (33.4)	297 (22.6)	46 (30.3)	261 (23.2)

For peer review only - http://bmjopen.bmj.com/site/about/guidelines.xhtml

Table 2: Characteristics of participants and child absenteeism, academic achievement and repetition of the school year

Exposure variables at age of 7/8 years		Child School absenteeism at end of 2014/15 academic year	Child Academic achievement at at end of 2014/15 academic year	Child grade repetition status at end of 2014/15 academic year	
		Median (25th, 75th percentiles)	Mean (SD)	Class Repeated	Promoted to next grade
		5 (2, 11)	62.5 (9.21)	N (%)	N (%)
				25 (2.4)	1029 (97.6)
**Parental characteristics**					
Maternal age in years (mean (SD))			-	34 (6.00)	34 (6.00)
Literacy (mother)	Non-literate	2 (5, 12)	62.5 (9.29)	22 (88.0)	900 (87.9)
Literacy (father)	Non-literate	3 (6, 14)	61.3 (9.23)	13 (54.2)	351 (35.8)
Maternal marital status	Monogamous	2 (5, 12)	62.8 (9.34)	19 (76.0)	824 (81.4)
	Polygamous	2 (4, 9)	61.1 (8.91)	5 (20.0)	140 (13.8)
	Divorced, widowed, separated	1 (4, 6)	62.1 (8.13)	1 (4.0)	48 (4.7)
**Socioeconomic status**					
Current hunger	Had hunger	2 (5, 11)	62.1 (7.75)	1 (4.0)	44 (4.3)
Emergency resource	Had no emergency resource	2 (5, 11)	62.1 (9.07)	9 (36.0)	288 (28.1)
Roof materials	Thatched	3 (6, 14)	62.8 (9.65)	17 (68.0)	524 (51.2)
**Psychosocial characteristics**					
Paternal substance use	Weekly khat use	2 (5, 12)	62.3 (9.24)	16 (66.7)	791 (82.4)
	Weekly alcohol use	1 (3.5, 7)	64.6 (9.42)	6 (25.0)	150 (15.6)
Negative life event in the last six months	No life event	2 (5, 12)	62.5 (9.14)	20 (80.0)	775 (75.6)
	1 life event	2 (4, 9)	62.8 (9.84)	3 (12.5)	180 (17.6)
	2 or more	2 (3, 8)	61.2 (8.21)	2 (8.0)	68 (6.6)
**Child characteristics**					
Childbirth order	First	2 (4.5, 14)	62.8 (8.87)	5 (20.0)	136 (13.3)
	Middle or last	2 (5, 11)	62.5 (9.26)	20 (80.0)	887 (86.7)
Sex of the child	Boy	2 (5, 12)	62.6 (9.23)	15 (60.0)	528 (51.6)
Child nutritional status	Stunted (Height-for-age z-score < -2)	2 (5, 12)	62.5 (9.20)	6 (24.0)	233 (23.0)

SD= standard deviation

School drop-out was 20.2% (n=336) at the end of the 2013/14 academic year. At the end of the 2014/15 academic year, drop-out was 11.8% (n=153), the median number of days of absence

For peer review only - http://bmjopen.bmj.com/site/about/guidelines.xhtml

was 5 (interquartile range 2 - 11), and the mean average grade point was 62.5 out of 100 (Standard Deviation 9.21). The correlation of SRQ20 score with absenteeism (Spearman's rank correlation coefficient = 0.008; p=0.777) and averaged grade point (r=-0.039; p=0.208) was not significant. In the 2013/14 academic year the median score of SRQ20 was 1, (interquartile range 0 – 2) in children who dropped out versus 0, (interquartile range 0- 1) in those who stayed in school (p=0.003; Mann-Whitney test). Similarly, in 2014/15 academic year, for in-school children the median SRQ20 score was 0, (interquartile range 0 – 2) versus 1, (interquartile range 0 – 3.5) in those dropped out children (p=0.02; Mann-Whitney test).

Controlling for potential confounders, maternal CMD at 7/8 years was associated significantly with school drop-out (OR 1.07, 95% CI: 1.00, 1.13) by the end of the 2014/15 academic year , but the significant unadjusted association between maternal CMD at 6/7 years and school drop-out by the end of the 2013/14 academic year became non-significant (adjusted OR 1.05, 95% CI: 0.99, 1.12). In the fully adjusted multivariable model, parent non-literacy, low socioeconomic status, male gender, not being first in the birth order and lower height-for-age were associated significantly with school drop-out by the end of the 2013/14 academic year. Paternal khat use and lower child height-for-age were associated significantly with 2014/15 academic year school drop-out. Maternal CMD at 7/8 years was associated with 2014/15 academic year school absenteeism (adjusted Incidence Rate Ratio 1.01, 95% CI: 1.00, 1.02). Absenteeism was also associated significantly with maternal age, parent non-literacy, widowed or separated marital status, low socioeconomic status, mother experiencing negative life event, child male gender, and being first in the birth order. See Tables 3 and 4.

For peer review only - http://bmjopen.bmj.com/site/about/guidelines.xhtml

Table 3: Factors associated with child school drop-out

**Exposure variables**	**Exposure variables at age of 6/7 years and school drop-out at end of 2013/14 academic year Odds Ratio (95% Confidence Interval)**		**Exposure variables at age of 7/8 years and school drop-out at 2014/15 academic years Odds Ratio (95% Confidence Interval)**	
	**Crude**	**Adjusted §**	**Crude**	**Adjusted §**
**Primary exposure**				
Maternal CMD (SRQ-20 total score)	**1.07 (1.02 ,1.13)**	1.05 (0.99 , 1.12)	**1.07 (1.01,1.13)**	**1.07 (1.00, 1.13)**
**Other explanatory variables**				
Maternal age (years)	**1.03 (1.01, 1.05)**	1.02 (0.99, 1.03)	1.01 (0.98, 1.04)	1.01 (0.97, 1.05)
Non-literate mother	**2.20 (1.46, 3.33)**	**1.77 (1.13, 2.77)**	1.94 (0.99, 3.76)	1.64 (0.82, 3.27)
Non-literate father	**1.75 (1.37, 2.25)**	**1.53 (1.17, 2.01)**	1.26 (0.89, 1.78)	1.19 (0.82, 1.74)
Maternal marital status (polygamous, divorced and widowed)	1.08 (0.99, 1.18)	1.07 (0.82, 1.43)	1.03 (0.91, 1.18)	1.18 (0.78, 1.78)
Experienced hunger due to lack of resources	**2.22 (1.50, 3.29)**	**1.74 (1.10, 2.77)**	1.85 (0.96, 3.56)	1.78 (0.82, 3.84)
No emergency resources	**1.65 (1.30, 2.11)**	**1.43 (1.10, 1.89)**	1.20 (0.83, 1.72)	1.05 (0.69, 1.60)
Thatched roof	**1.65 (1.27, 2.14)**	1.30 (0.96, 1.74)	**1.43 (1.01, 2.03)**	1.33 (0.91, 1.96)
Paternal khat use at least weekly	**1.52 (1.10, 2.11)**	1.38 (0.98, 1.95)	**0.54 (0.37, 0.81)**	**0.52 (0.34, 0.79)**
Paternal alcohol use at least weekly	1.23 (0.89, 1.71)	1.35 (0.95, 1.93)	1.35 (0.88, 2.09)	1.18 (0.73, 1.89)
Negative life event (≥1 in the last 6 months)	0.99 (0.84, 1.16)	0.85 (0.69, 1.03)	1.04 (0.98, 1.89)	1.00 (0.77, 1.29)
Male child	**1.45 (1.14, 1.85)**	**1.50 (1.15, 1.96)**	1.15 (0.82, 1.62)	1.13 (0.78, 1.63)
Child first in birth order	**0.44 (0.29, 0.67)**	**0.59 (0.36, 0.95)**	0.73 (0.43, 1.27)	0.82 (0.45, 1.51)
Height for age z score	**0.73 (0.65, 0. 83)**	**0.80 (0.70, 0.91)**	**0.75 (0.62, 0.89)**	**0.73 (0.60, 0.89)**

CMD = common mental disorder; SRQ = Self-Reporting Questionnaire;
§ Parental characteristics including (maternal age, marital status, maternal and paternal level of literacy), Socio-economic status (hunger due to lack of resources, emergency resources, roof material), paternal substance use, negative life event, child sex, birth order and child nutritional status

For peer review only - http://bmjopen.bmj.com/site/about/guidelines.xhtml

Table 4: Factors associated with child school absenteeism

Exposure variables at age 7/8 years of the child	School absenteeism at the end of 2014/15 academic years Incidence Rate Ratio (95% Confidence Interval)	
	Crude	Adjusted §
**Primary exposure**		
Maternal CMD (SRQ-20 total score)	1.00 (0.99, 1.01)	**1.01 (1.00, 1.02)**
**Other explanatory variables**		
Increasing maternal age (years)	1.00 (0.99, 1.01)	**1.01 (1.01, 1.02)**
Mother non literate	**1.60 (1.48, 1.74)**	**1.57 (1.44, 1.72)**
Father non literate	**1.18 (1.13, 1.23)**	**1.07 (1.02, 1.12)**
Marital status (polygamous, divorced, widowed)	**0.92 (0.89, 0.94)**	**0.88 (0.83, 0.93)**
Experienced hunger due to lack of resources	1.06 (0.96, 1.17)	1.06 (0.94, 1.19)
No emergency resources	1.03 (0.99, 1.08)	0.98 (0.94, 1.04)
Thatched roof (vs. corrugated iron)	**1.46 (1.40, 1.53)**	**1.36 (1.30, 1.42)**
Father uses khat at least weekly	**1.10 (1.03, 1.17)**	1.04 (0.97, 1.11)
Father uses alcohol at least weekly	**0.90 (0.85, 0.96)**	0.92 (0.86, 0.98)
Negative life event (≥1 in the last 6 months)	**0.98 (0.97, 0.99)**	**0.90 (0.87, 0.94)**
Male child	1.04 (1.00, 1.09)	**1.05 (1.00, 1.09)**
Child first in birth order	**1.14 (1.08, 1.21)**	**1.17 (1.10, 1.25)**
Height for age z score	1.00 (0.97, 1.01)	1.00 (0.97, 1.02)

CMD = common mental disorder; SRQ = Self-Reporting Questionnaire;

§ Parental characteristics including (maternal age, marital status, maternal and paternal level of literacy), Socio-economic status (hunger due to lack of resources, emergency resources, roof material), paternal substance use, negative life event, child sex, birth order and child nutritional status

There was no significant association between maternal CMD and academic achievement or grade repetition. Paternal non-literacy, alcohol use and widowed or separated maternal marital status were significantly associated independently with these outcomes. See Table 5.

For peer review only - http://bmjopen.bmj.com/site/about/guidelines.xhtml

Table 5: Factors associated with child academic achievement

CMD = common mental disorder; SRQ = Self-Reporting Questionnaire;

Exposure variables at age of 7/8 years of the child	Academic achievement by end of 2014/15 academic year β coefficient (95% Confidence Interval)		Grade repetition by end of 2014/15 academic year Odds Ratio (95% Confidence Interval)	
	Crude	Adjusted §	Crude	Adjusted §
Primary exposure				
Maternal CMD (SRQ-20 total score)	-0.07 (-0.27, 0.13)	0.01 (-0.22, 0.23)	1.01 (0.87, 1.16)	0.99 (0.85, 1.16)
Other explanatory variables				
Maternal age	-0.03 (-0.13, 0.08)	0.01 (-0.11, 0.13)	1.01 (0.94, 1.09)	1.05 (0.96, 1.14)
Mother non-literate	0.21 (-1.51, 1.92)	0.46 (-1.42, 2.34)	1.00 (0.29, 0.97)	0.87 (0.24, 3.17)
Father non- literate	**-1.88 (-3.06, -0.69)**	**-1.90 (-3.16, -0.64)**	2.12 (0.93, 4.78)	1.89 (0.80, 4.44)
Marital status (polygamous, divorced and widowed)	-0.21 (-0.67, 0.26)	**-2.02 (-3.48, -0.55)**	0.96 (0.67, 1.38)	1.31 (0.54, 3.16)
Experienced hunger due to lack of resources	-0.42 (-3.18, 2.33)	-0.79 (-4.14, 2.56)	0.93 (0.12, 7.00)	0.97 (0.11, 8.74)
No emergency resources	-0.59 (-1.83, 0.64)	-0.59 (-2.00, 0.80)	1.43 (0.63, 3.28)	1. 50 (0.60, 3.74)
Thatched roof	0.54 (-0.57, 1.66)	0.78 (-0.46, 2.00)	2.02 (0.86, 4.73)	1.55 (0.62, 3.89)
Father uses khat at least weekly	-1.31 (-2.82, 0.21)	-0.57 (-2.15, 1.00)	0.43 (0.18, 1.01)	0.44 (0.18, 1.12)
Father uses alcohol at least weekly	**2.47 (0.88, 4.05)**	**2.35 (0.65, 4.04)**	1.80 (0.70, 4.62)	1. 26 (0.45, 3.51)
Negative life event in the last six months	-0.07 (-0.33, 0.18)	-0.24 (-1.09, 0.61)	1.04 (0.90, 1.18)	0.76 (0.34, 1.72)
Male child	0.16 (-0.95, 1.28)	-0.09 (-1.10, 1.27)	1.41 (0.63, 3.16)	1.45 (0.61, 3.42)
Child first in birth order	0.34 (-1.30, 1.97)	0.33 (-1.50, 2.17)	1.63 (0.60, 4.41)	2.09 (0.66, 6.60)
Height for age z score	**0.62 (0.00, 1.23)**	0.59 (-0.07, 1.25)	1.08 (0.69, 1.67)	1.12 (0.69, 1.81)

§ Parental characteristics including (maternal age, marital status, maternal and paternal level of literacy), Socio-economic status (hunger due to lack of resources, emergency resources, roof material), paternal substance use, negative life event, child sex, birth order and child nutritional status

For peer review only - http://bmjopen.bmj.com/site/about/guidelines.xhtml

DISCUSSION

We found a significant prospective association between maternal CMD and child school drop-out and absenteeism. The unadjusted association between exposure to maternal CMD at age 6/7 years and drop-out by the end of the 2013/14 academic year became non-significant when adjusted for potential confounding variables. Parental low socioeconomic status, non-literacy and substance use were associated significantly with adverse educational outcomes of children. There was no association between maternal CMD and child academic achievement or grade repetition.

As far as we are aware, our study is the first of its kind from a low-income country to report the association between exposure to preschool maternal CMD and subsequent adverse educational outcomes. Strengths of the study included the population-based design, large sample size, high follow-up rates and use of culturally validated measures. Nonetheless, there were limitations of our study. Maternal CMD was assessed using a symptom screening scale rather than a diagnostic measure. We assessed academic achievement using a composite and non-standardised measure (individual teacher assessment). Although this approach increased measurement error, composite measures which include participation, homework, attendance and tests given by teachers may be more ecologically valid and tied to day-to-day routine of teaching and learning than narrowly focused assessments of content mastery. Absenteeism was extracted from data collected routinely by schools, which is likely to have led to underestimation of absences, but this would have led to a non-differential error which would be expected to reduce the chance of finding a significant association and would not be

For peer review only - http://bmjopen.bmj.com/site/about/guidelines.xhtml

expected to have led to bias. We relied on proxy indicators of socioeconomic status. Although these indicators have been developed for the population under study, they relied on self-report from the women and may not have been sufficiently comprehensive, thus raising the possibility of residual confounding. Physical ill-health of the child may also have confounded the association.

The higher school drop-out rate at the end of 2013/14 academic year over the subsequent year is in keeping with existing evidence. The non-significant association between maternal CMD and academic achievement and grade repetition in our study is in contrast to the study from Barbados, where postpartum depression was associated with poorer academic achievement at 11 years of age.[20] This difference may have arisen due to the measures of academic achievement used (standardized in Barbados vs. subjective/composite measure in Ethiopia) and the timing of the CMD exposure (postpartum vs. pre- and early school years). Furthermore, the broader context for learning also differs substantially between Barbados and Ethiopia. In Ethiopia, the majority of women are non-literate and there is limited availability of learning resources (e.g. books, games) within the home. Given the critical importance of these factors for child learning, any additional effect of maternal CMD may have been too small to detect, unlike in Barbados.

On the other hand, the deficits in the home learning environment mean that regular attendance at school becomes the most important way for a child to learn and is likely to have greater influence on child educational performance. Our findings were in keeping with the

For peer review only - http://bmjopen.bmj.com/site/about/guidelines.xhtml

Barbados study, where depressive symptoms in mothers of a child with an early history of malnutrition were associated with school absences between 5 and 11 years of age. Irregular school attendance is likely to be on the pathway to poorer educational attainment and to identify a group of children who are at risk of fully dropping out from school.

In studies from HICs, erratic school attendance and drop-out from school have been associated with an adverse early home environment, living with smokers, the quality of early care-giving, socioeconomic status, cognitive development, behavior problems, academic achievement, peer relations and parent involvement.[32, 33] In LMICs, food insecurity and death of parents[34, 35] are associated with irregular school attendance and dropping out entirely. In both HIC and LMIC studies, maternal CMD could be an important unmeasured mediator of the effects of adverse home environment on school attendance. For children under the age of ten years, absenteeism and drop-out are unlikely to happen without the knowledge of the family, and in particular the mother.

School absenteeism and drop-out have important economic implications, particularly in a low-income country like Ethiopia. Repeating years of education or students taking longer to complete their education bring inefficiency into the school system, financial burden on the household and may adversely affect the motivation of the child to pursue their education. For those students who drop-out of school altogether, there are economic losses from negative impacts on future productivity. Every additional year of schooling has a substantial positive effect on adult wages.[36] Achieving high coverage of primary school enrolment is undermined

For peer review only - http://bmjopen.bmj.com/site/about/guidelines.xhtml

by subsequent early drop-out from schooling, which will limit Ethiopia's ability to deliver on the Sustainable Development Goal (SDG) target to ensure that all girls and boys complete free, equitable and quality primary and secondary education by 2030.

School-based interventions targeting specific predictors of absenteeism and drop-out in LMICs, e.g. deworming,[37] hand wash campaigns, safe water and hygiene,[38] and school feeding programmes,[39] have been found to be effective in reducing absenteeism and drop-out. Our study indicates that interventions (at family, school or community level) focusing on the reduction of absenteeism and drop-out need also to incorporate maternal CMD. There is a global impetus to improve access to primary care-based mental health care in LMICs through the World Health Organisation's mental health Gap Action Programme.[40] Evidence-based treatment packages for maternal depression that can be delivered by general health workers (assessment and prescription of medication) and lay workers (psychosocial approaches) have been shown to be effective, feasible and acceptable to communities across LMIC settings.[41] Effective care for maternal depression has been shown to have beneficial effects on child health in Pakistan[42] but further studies are needed to evaluate the impact on child educational outcomes.

For peer review only

CONCLUSION

In this population-based cohort study in rural Ethiopia, children exposed to mothers with symptoms of common mental disorders during pre and early school age were at greater risk of school drop-out and absenteeism. Future studies are needed to understand the mechanisms underlying this association. Current global efforts to expand access to mental health care offer

For peer review only - http://bmjopen.bmj.com/site/about/guidelines.xhtml

an opportunity to address maternal CMD as a key component of programmes to increase school attendance.

Acknowledgements

We are grateful for the women who participated in the study for giving their time and energy to complete interviews and to the staff in the schools who facilitated data collection process. Our thanks also go to the project data collectors and staff of the C-MaMiE project in Butajira.

Contributors

HM, CH, AA and MP conceptualised the study. HM led data collection with oversight from CH, AA and GM. HM led data analysis, with input from CH, AA, MP, MT and GM. HM drafted the manuscript and all other co-authors revised it critically. All authors approved the final version of the paper and agree to be accountable for all aspects of the work.

Funding

The funding for the study came from the Wellcome Trust (project grant: 093559).

Competing interests

The funding for the study came from the Wellcome Trust.

For peer review only - http://bmjopen.bmj.com/site/about/guidelines.xhtml

Ethical approval

Ethical approval was obtained from the Institutional Review Board of the College of Health Sciences, Addis Ababa University, Ethiopia (reference number 082/13/psy) and the Research Ethics Committee of King's College London, UK (reference number PNM/13/14-92).

Data sharing statement

The data are being used for a PhD student (HM) for his thesis and are not, therefore, available at the present time to the general public. The data may be requested from the corresponding author for verification of the analyses in this paper.

Figure 1: Conceptual framework

Figure 2: Follow-up chart for child educational outcomes

For peer review only - http://bmjopen.bmj.com/site/about/guidelines.xhtml

For peer review only

REFERENCES

1. United Nations, *Convention on the Rights of the Child*. 1989, UN: Geneva.
2. Goldberg. D and H. P., *Common mental disorders: a biosocial model. London: Routledge; 1992.* 1992.
3. Fisher, J., et al., *Prevalence and determinants of common perinatal mental disorders in women in low- and lower-middle-income countries: a systematic review:.* Bull World Health Organ ;90:139–149G | doi:10.2471/BLT.11.091850:, 2012.
4. Galler, J.R., et al., *Maternal depressive symptoms affect infant cognitive development in Barbados.* Journal of Child Psychology & Psychiatry & Allied Disciplines, 2000. **41**(6): p. 747-757.
5. Patel, V., N. DeSouza, and M. Rodrigues, *Postnatal depression and infant growth and development in low income countries: a cohort study from Goa, India.* Archives of Disease in Childhood, 2003. **88**(1): p. 34-7.
6. Black, M.M., et al., *Maternal depressive symptoms and infant growth in rural Bangladesh.* The American Journal of Clinical Nutrition, 2009. **89**(3): p. 951S-957S.
7. Harpham, T., et al., *Maternal mental health and child nutritional status in four developing countries.* Journal of Epidemiology and Community Health, 2005. **59**: p. 1060-1064.
8. Cooper, P.J., et al., *Improving quality of mother-infant relationship and infant attachment in socioeconomically deprived community in South Africa: randomised controlled trial.* BMJ, 2009. **338**: p. b974.
9. Servili, C., et al., *Maternal common mental disorders and infant development in Ethiopia: the P-MaMiE Birth Cohort.* BMC Public Health, 2010. **10**: p. 693.
10. Cooper, P.J., et al., *Post-partum depression and the mother-infant relationship in a South African peri-urban settlement.* British Journal of Psychiatry, 1999. **175**: p. 554-8.
11. Murray, L., et al., *The impact of postnatal depression and associated adversity on early mother-infant interactions and later infant outcome.* Child Development, 1996. **67**(5): p. 2512-26.
12. Ferri, C.P., et al., *The impact of maternal experience of violence and common mental disorders on neonatal outcomes: a survey of adolescent mothers in Sao Paulo, Brazil.* BMC Public Health, 2007. **7**(1): p. 209.
13. Hanlon, C., et al., *Impact of antenatal common mental disorders upon perinatal outcomes in Ethiopia: the P-MaMiE population-based cohort study.* Trop Med Int Health, 2009. **14**(2): p. 156-66.
14. Rahman, A., et al., *Impact of maternal depression on infant nutritional status and illness: a cohort study.* Archives of General Psychiatry, 2004. **61**(9): p. 946-52.
15. Adewuya, A.O., et al., *Impact of postnatal depression on infants' growth in Nigeria.* Journal of Affective Disorders, 2008. **108**(1-2): p. 191-3.
16. Surkan, P., et al., *Maternal depression and early childhood growth in developing countries: systematic review and meta-analysis.* Bull World Health Organ 2011. **287**: p. 607-615.
17. Ramchandani, P., et al., *Paternal depression in the postnatal period and child development: a prospective population study.* Lancet, 2005. **365**: p. 2201-2205.
18. Parsons, C.E., et al., *Postnatal depression and its effects on child development: a review of evidence from low- and middle-income countries.* British Medical Bulletin, 2012. **101**: p. 57-79.

For peer review only - http://bmjopen.bmj.com/site/about/guidelines.xhtml

19. Salt, P., J.R. Galler, and C.F. Ramsey, *The influence of early malnutrition on subsequent behavioural development. VII. The effect of maternal depressive symptoms.* Developmental and Behavioural Pedatrics, 1988. **9**(1): p. 1-5.
20. Galler, J.R., et al., *Postpartum maternal moods and infant size predict performance on a national high school entrance examination.* Journal of Child Psychology & Psychiatry, 2004. **45**(6): p. 1064-1075.
21. Berhane, Y., et al., *Establishing an epidemiological field laboratory in rural areas - potentials for public health research and interventions (Special Issue).* Ethiopian Journal of Health Development, 1999. **13**.
22. Federal Democratic Government of Ethiopia Ministry of Education, *The education and training policy and its implementation, Ethiopia*. 2002: Addis Ababa, Ethiopia,.
23. Federal Democratic Government of Ethiopia Ministry of Education, *Education Statistics 2007 E.C. (2014/2015)*, in *Education Statistics Annual Abstract*. 2016, MoE, EMIS and ICT Directorate,: Addis Ababa, Ethiopia.
24. Beusenberg, M. and J. Orley, *A user's guide to the Self Reporting Questionnaire (SRQ)*. 1994, World Health Organization: Geneva.
25. Hanlon, C., et al., *Detecting perinatal common mental disorders in Ethiopia: validation of the self-reporting questionnaire and Edinburgh Postnatal Depression Scale.* J Affect Disord, 2008. **108**(3): p. 251-62.
26. Brugha, T.S., et al., *The list of threatening experiences: a subset of 12 life event categories with considerable long-term contextual threat.* Psychological Medicine, 1985. **15**: p. 189-194.
27. WHO Expert Committee, *Physical status: the use and interpretation of anthropometry*, in *WHO Technical Report Series 854*. 1995, WHO: Geneva.
28. World Health Organization, *Anthro software version 3.2.2*, in http://www.who.int/childgrowth/software/en/. 2011, WHO: Geneva.
29. Keller, W. and C.M. Fillmore, *Prevalence of protein-energy malnutrition.* World Health Star Quarterly, 1983. **36**: p. 129.
30. Lauritsen, J.M. and M. Bruus, *Epidata (Version 3). A comprehensive tool for validated entry and documentation of data.* . 2003, The Epidata Association,: Odense, Denmark,.
31. Stata Corporation, *Stata Statistical Software Release 12*. 2007, Stata Corporation: College Station, TX,.
32. Jimerson, S., et al., *A prospective longitudinal study of high school dropouts: examining multiple predictors across development.* Journal of School Psychology, 2000. **38**(6): p. 525-549.
33. Levy, D.E., J.P. Winickoff, and N.A. Rigotti, *School absenteeism among children living with smokers.* Pediatrics, 2011. **128**(4): p. 650-656.
34. Case, A. and C. Ardington, *The impact of parental death on school outcomes: longitudinal evidence from South Africa.* Demography, 2006. **43**(3): p. 401-420.
35. Belachew, T., et al., *Food insecurity, school absenteeism and educational attainment of adolescents in Jimma Zone Southwest Ethiopia: a longitudinal study.* Nutrition Journal, 2011. **10**: p. 29.
36. Day, J.C. and E.C. Newburger, *The big payoff: educational attainment and synthetic estimates of work-life earnings. Special studies.* , in *Current Population Reports*. 2002, Bureau of the Census (DOC), Economics and Statistics Administration: Washington, DC,. p. Accessd from: http://www.census.gov/prod/2002pubs/p23-210.pdf on 11th April 2017.

For peer review only - http://bmjopen.bmj.com/site/about/guidelines.xhtml

37. Aiken, M.A., et al., *Re-analysis of health and educational impacts of a school-based deworming programme in western Kenya: a pure replication.* International Journal of Epidemiology, 2015: p. 1572-1580 doi: 10.1093/ije/dyv127.
38. O'Reilly, C.E., et al., *The impact of a school-based safe water and hygiene programme on knowledge and practices of students and their parents: Nyanza Province, western Kenya, 2006.* Epidemiol Infect, 2008. **136**(1): p. 80-91.
39. King, N., C. Dewey, and D. Borish, *Determinants of primary school non-enrollment and absenteeism: results from a retrospective, convergent mixed methods, cohort study in rural western Kenya.* PLoS One, 2015. **10**(9): p. e0138362.
40. World Health Organization, *mhGAP: Mental Health Gap Action Programme: scaling up care for mental, neurological, and substance use disorders*. 2008, WHO: Geneva.
41. World Health Organization, *mhGAP Intervention Guide version 2.0 for mental, neurological and substance use disorders in non-specialized health settings*. 2016, WHO: Geneva.
42. Maselko, J., et al., *Effect of an early perinatal depression intervention on long-term child development outcomes: follow-up of the Thinking Healthy Programme randomised controlled trial.* The Lancet Psychiatry, 2015. **2**(7): p. 609-617.

For peer review only

For peer review only - http://bmjopen.bmj.com/site/about/guidelines.xhtml

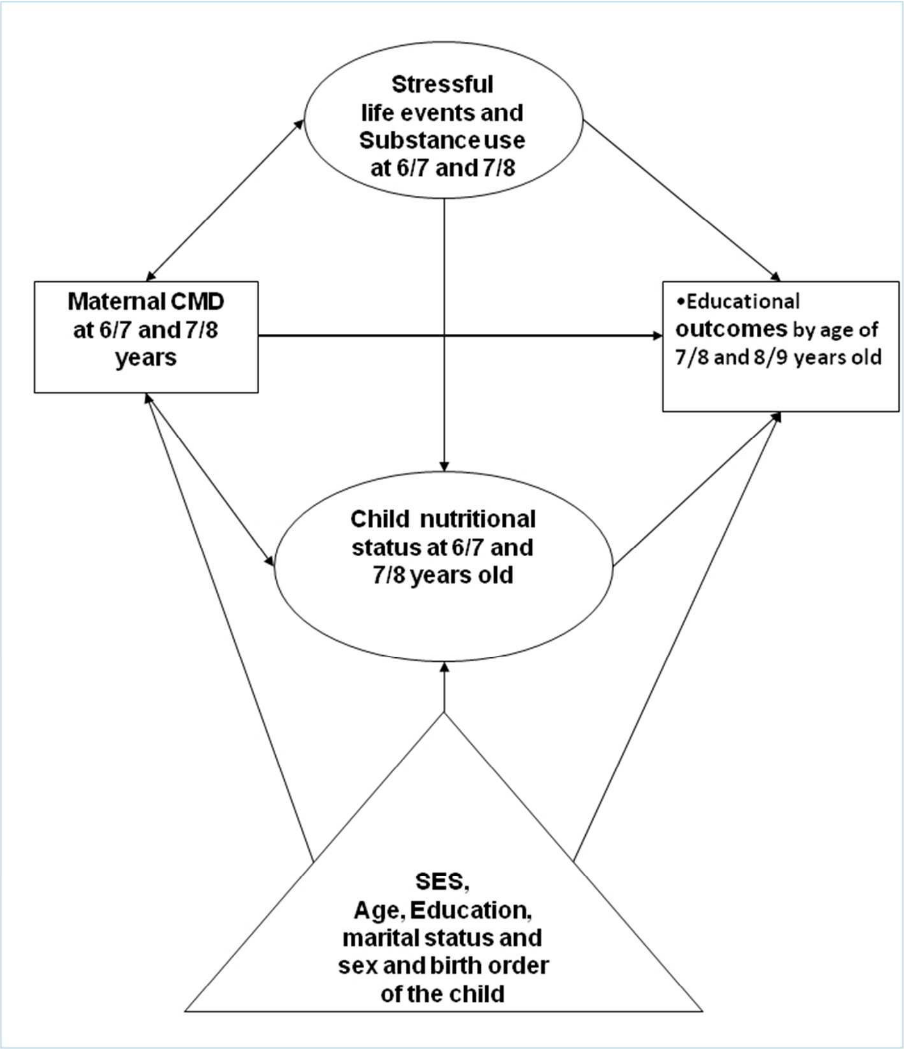

Figure 1: Conceptual framework

65x81mm (300 x 300 DPI)

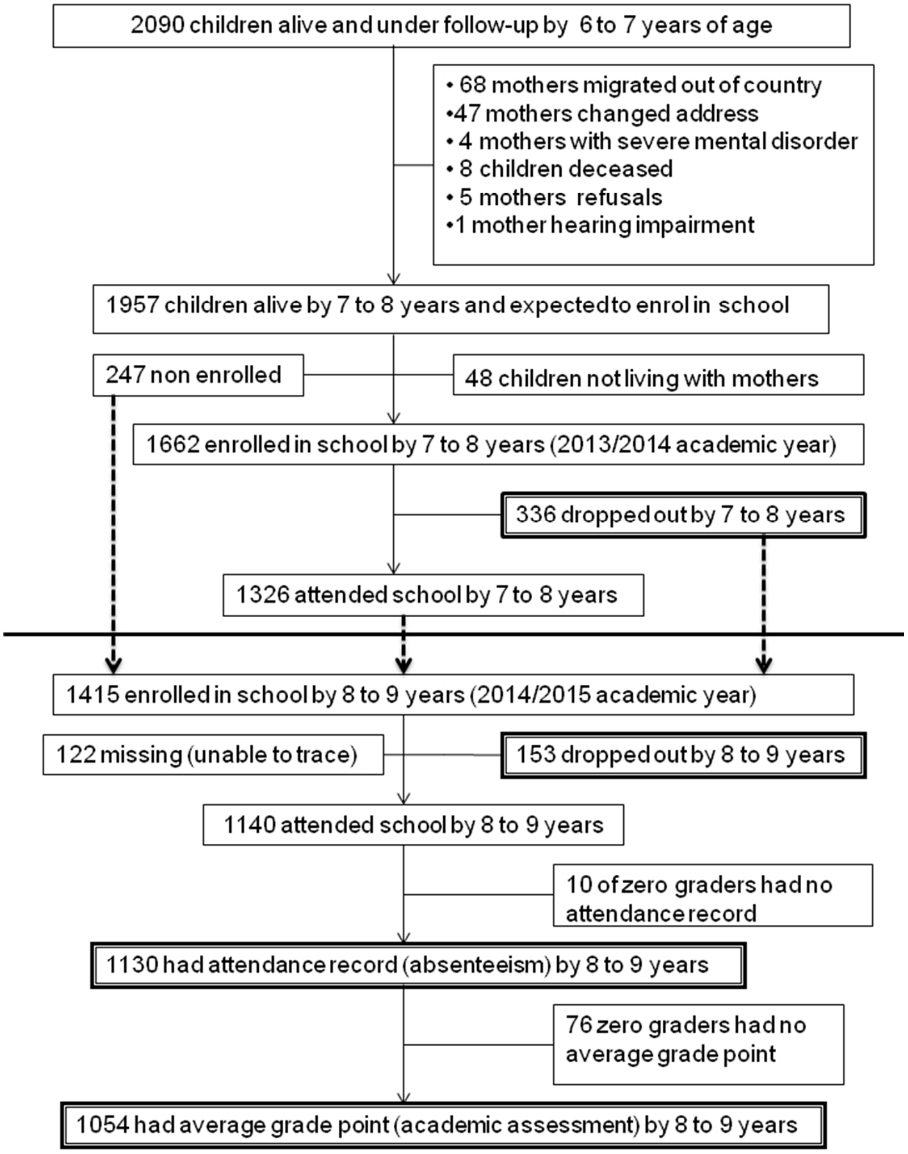

Figure 2: Flow chart of study participants

60x81mm (300 x 300 DPI)
